# Human CD127 negative ILC2s show immunological memory

**DOI:** 10.1084/jem.20231827

**Published:** 2024-06-18

**Authors:** Laura Mathä, Lisette Krabbendam, Sergio Martinez Høyer, Balthasar A. Heesters, Korneliusz Golebski, Chantal Kradolfer, Maryam Ghaedi, Junjie Ma, Ralph Stadhouders, Claus Bachert, Lars-Olaf Cardell, Nan Zhang, Gabriele Holtappels, Sietze Reitsma, Leanne Carijn Helgers, Teunis B.H. Geijtenbeek, Jonathan M. Coquet, Fumio Takei, Hergen Spits, Itziar Martinez-Gonzalez

**Affiliations:** 1 Microbiology, Tumor and Cell Biology, Karolinska Institute, Stockholm, Sweden; 2 Terry Fox Laboratory, British Columbia Cancer, Vancouver, Canada; 3 Department of Experimental Immunology, Amsterdam University Medical Center, University of Amsterdam, Amsterdam, Netherlands; 4 Department of Pulmonary Medicine, Erasmus Medical Center, University of Rotterdam, Rotterdam, Netherlands; 5 Chemical Biology and Drug Discovery, Utrecht Institute for Pharmaceutical Sciences, Faculty of Science, Utrecht University, Utrecht, Netherlands; 6 Department of Pulmonary Medicine, Amsterdam University Medical Center, Amsterdam, Netherlands; 7 Princess Margaret Cancer Centre, University Health Network, Toronto, Canada; 8 Department of Oto-Rhino-Laryngology, Münster University, Münster, Germany; 9 Sun Yat-sen University, The First Affiliated Hospital, Guangzhou, China; 10 Upper Airway Research Laboratory, Ghent University, Ghent, Belgium; 11 ENT-Department, Karolinska University Hospital, Stockholm, Sweden; 12 Department of Otorhinolaryngology, Karolinska University Hospital, Stockholm, Sweden; 13 Department of Otorhinolaryngology and Head/Neck Surgery, University of Amsterdam, Amsterdam University Medical Center, Amsterdam, Netherlands; 14 Amsterdam Institute for Infection & Immunity, Amsterdam University Medical Center, University of Amsterdam, Amsterdam, Netherlands; 15 Department of Pathology and Laboratory Medicine, University of British Columbia, Vancouver, Canada

## Abstract

ILC2s are key players in type 2 immunity and contribute to maintaining homeostasis. ILC2s are also implicated in the development of type 2 inflammation–mediated chronic disorders like asthma. While memory ILC2s have been identified in mouse, it is unknown whether human ILC2s can acquire immunological memory. Here, we demonstrate the persistence of CD45RO, a marker previously linked to inflammatory ILC2s, in resting ILC2s that have undergone prior activation. A high proportion of these cells concurrently reduce the expression of the canonical ILC marker CD127 in a tissue-specific manner. Upon isolation and in vitro stimulation of CD127^−^CD45RO^+^ ILC2s, we observed an augmented ability to proliferate and produce cytokines. CD127^−^CD45RO^+^ ILC2s are found in both healthy and inflamed tissues and display a gene signature of cell activation. Similarly, mouse memory ILC2s show reduced expression of CD127. Our findings suggest that human ILC2s can acquire innate immune memory and warrant a revision of the current strategies to identify human ILC2s.

## Teaser

Mathä et al. report a resting CD127^−^CD45RO^+^ ILC2 population in humans that exhibits features of memory cells. These memory ILC2s are present in the circulation and tissues of healthy individuals and patients with chronic type 2 inflammation.

## Graphical Abstract

**Figure:**
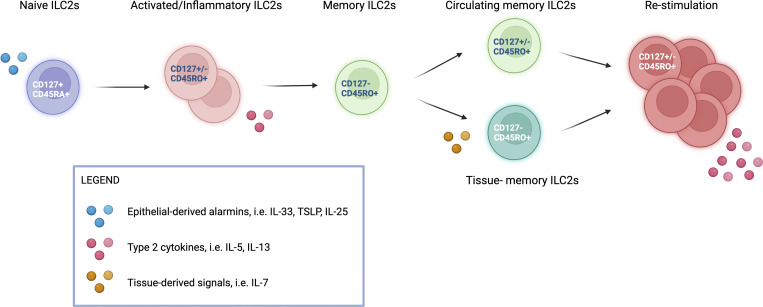

## Introduction

Group 2 innate lymphoid cells (ILC2s) are abundant in barrier tissues in both mice and humans and are initiators of immune responses against allergens and parasites (Moro et al., 2010; Neill et al., 2010; Price et al., 2010). ILC2s are devoid of markers that define lineages such as T, B, and myeloid cells but express IL-7Rα (CD127), IL-2Rα (CD25), the receptor for prostaglandin D2 (CRTH2) (Mjösberg et al., 2011), and inducible T cell costimulator (ICOS) as well as the transcription factors GATA3 (Hoyler et al., 2012; Mjösberg et al., 2012) and retinoic acid receptor-related orphan receptor α (RORA) (Halim et al., 2012). ILC2s are mainly tissue resident (Gasteiger et al., 2015) and are activated by epithelial- or stromal-derived alarmins including the cytokines IL-33, IL-25, and thymic stromal lymphopoietin (TSLP) (Krabbendam et al., 2018a). In response, they are capable of producing type 2 cytokines, including IL-4, IL-5, and IL-13, initiating an inflammatory cascade that leads to type 2 inflammation characterized by eosinophilia, mucus production, and epithelial barrier leakage (Liu et al., 2015; Martinez-Gonzalez et al., 2015). Consequently, ILC2s have been implicated in various type 2 inflammation–mediated chronic diseases, and higher numbers are found in inflamed tissues, such as the skin of atopic dermatitis (AD) patients (Kim et al., 2013), or nasal polyps from chronic rhinosinusitis with nasal polyps (CRSwNP) patients, compared with healthy tissues (Bal et al., 2016).

We previously described immunological memory of ILC2s in a mouse model of lung inflammation (Martinez-Gonzalez et al., 2016). Upon activation by IL-33 or allergens, ILC2s undergo an expansion phase marked by proliferation and IL-5 and IL-13 production. This is followed by a contraction phase where they stop producing cytokines but live for a long time. Upon secondary challenge by an unrelated allergen, previously activated ILC2s respond faster and more potently compared with naive ILC2s, leading to an enhanced inflammation. Whether human ILC2s can acquire memory functions remains unknown. We have recently shown that the expression of CD45RO marks inflammatory ILC2s in humans (van der Ploeg et al., 2021). Inflammatory ILC2s were highly activated and abundant in inflamed tissues such as nasal polyps from CRSwNP patients. CD45 is a receptor tyrosine phosphatase found on the surface of most blood cell types, including ILCs and T cells (Bunce and Bell, 1997). Whereas naive T cells express the CD45RA splicing isoform, activated and memory T cells exclusively express CD45RO. Inhibition of the activity of CD45 sensitizes ILC2s to cytokine production, suggesting that this phosphatase is implicated in the regulation of activation of CD45RA^+^ ILC2s (van der Ploeg et al., 2021).

We hypothesized that previously activated ILC2s could retain CD45RO expression and acquire memory features. Considering that humans are constantly exposed to extrinsic insults/stimuli, we suspected that memory ILC2s would be present in healthy individuals. CD127 is currently the canonical marker to identify ILCs in humans serving as a universal reference in the ILC field in various tissues and diverse disease contexts. Here, we show that human tissues contain a subset of CRTH2^+^ ILC2s that do not express CD127 and express high levels of CD45RO. CD127^−^CD45RO^+^ ILC2s are more responsive to epithelial alarmins than CD127^+^CD45RA^+^ ILC2s. Our data suggest that naive ILC2s, upon activation, modulate CD127, switch from CD45RA^+^ to CD45RO^+^, and persist as memory ILC2s that are highly responsive to secondary challenges.

## Results

### CD127^−^ ILC2s in inflamed tissues present an enhanced cell activation profile

**Figure S1.**
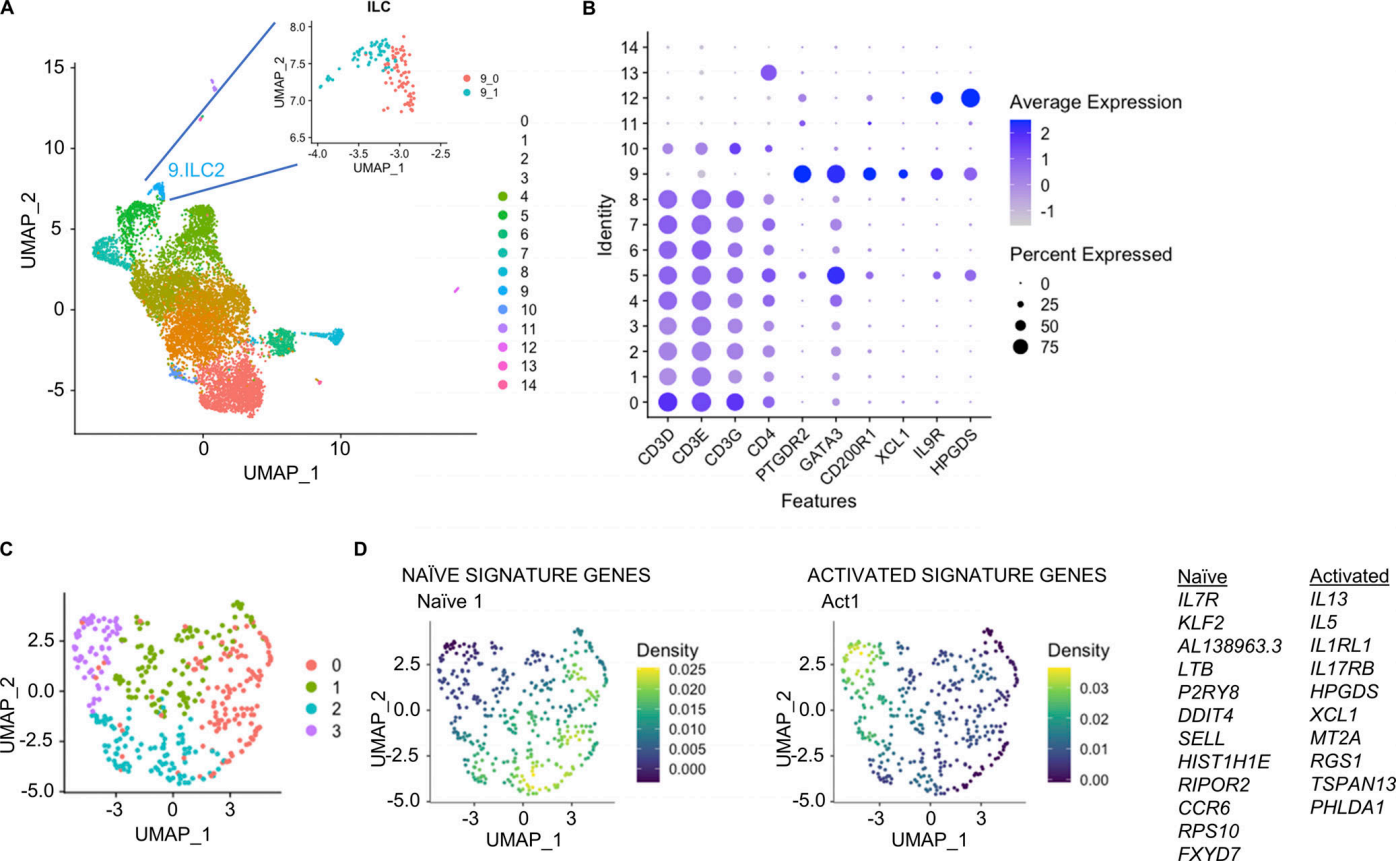
***IL7R*-low ILC2s are present in inflammed tissues. (A)** UMAP visual representation of the T and ILC2 (Lin^−^CRTH2^+^) clusters obtained from the analysis of the scRNAseq dataset from nasal polyps from CRSwNP patients (Ma et al., 2021). **(B)** Dot plot showing featured genes for each cluster identified in A. **(C)** UMAP visual representation of the cell clusters obtained from the analysis of the scRNAseq dataset from AD biopsies (Alkon et al., 2022). **(D)** Density plots of signature genes of naive and activated ILC2s from the scRNAseq dataset from AD biopsies.

**Figure 1.**
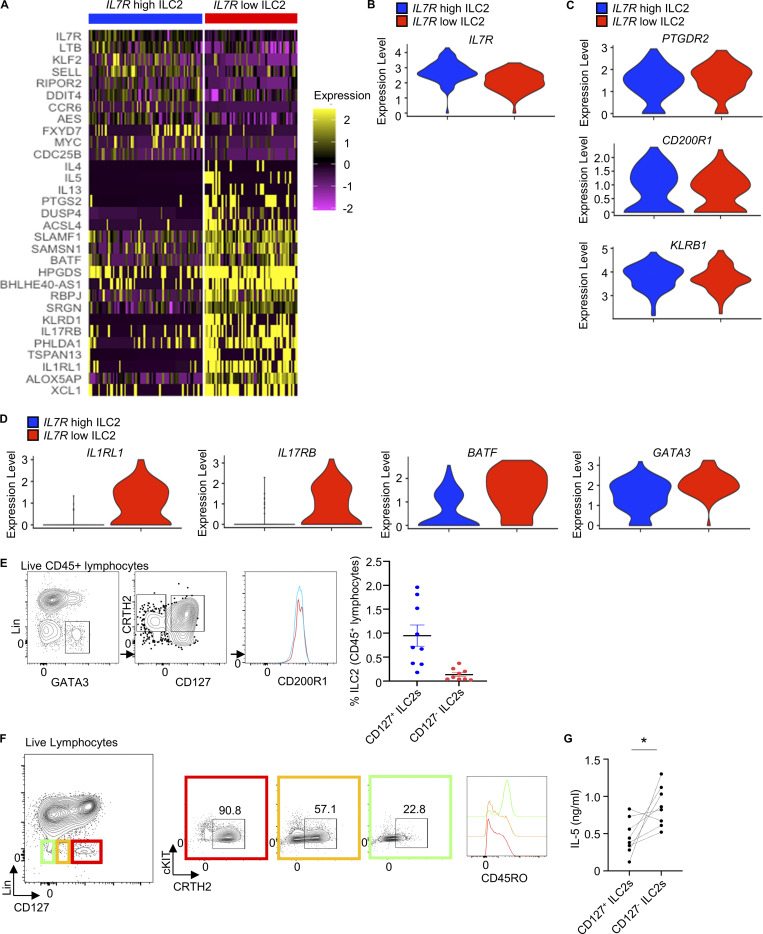
**CD127^−^ ILC2s in inflamed tissues present an enhanced cell activation profile. (A)** Heatmap of top marker genes from the CD127^+^ and CD127^−^ ILC2 clusters observed in the scRNAseq dataset from nasal polyps from CRSwNP patients (Ma et al., 2021). **(B)** Violin plot of *IL7R* expression in the CD127^+^ and CD127^−^ ILC2 clusters. **(C)** Violin plots of *PTGDR2* (top), *CD200R1* (middle), and *KLRB1* (bottom) expression in the CD127^+^ and CD127^−^ ILC2 clusters. **(D)** Violin plots of *ILRL1*, *IL17RB*, *BATF*, and *GATA3* expression in the CD127^+^ and CD127^−^ ILC2 clusters. **(E)** Gating strategy and quantification of CD127^+^ and CD127^−^ ILC2s (as Lineage-negative CRTH2^+^ cells, all of which expressed CD200R1) in fresh nasal polyps from CRSwNP patients analyzed by flow cytometry (*n* = 9). **(F)** CD45RO expression on CD127^+^ and CD127^−^ ILC2s in fresh nasal polyps from CRSwNP patients analyzed by flow cytometry. **(G)** IL-5 levels in the culture supernatant of CD127^+^ and CD127^−^ ILC2s isolated from nasal polyps, upon stimulation with IL-2+IL-7+IL-33+TSLP for 7 days measured by ELISA (*n* = 8). scRNAseq data analysis was performed using Seurat. FACS plots in E and F are representative of more than three donors from at least two independent experiments. Symbols represent individual donors from at least two independent experiments. *P ≤ 0.05 (paired *t* test).

Ma et al. recently published single-cell RNA sequencing (scRNAseq) data on nasal polyps from asthma patients with CRSwNP (Ma et al., 2021). ILC2s were isolated as live CD45^+^CD3^−^Lin^−^CRTH2^+^ cells and clustered together (cluster 9, Fig. S1 A) with a gene expression signature of *PTGDR2*, *GATA3*, *CD200R1*, *XCL1*, *IL9R*, and *HPGDS* (Fig. S1 B). Unsupervised clustering with a higher resolution further divided ILC2s into two clusters (Fig. 1 A and Fig. S1 A), which were defined by differential expression of *IL7R* among other marker genes (P adj = 0.0065) (Fig. 1 B). Therefore, we referred to these clusters as *IL7R*-hi and *IL7R*-low ILC2s. Both clusters showed equal expression of common ILC2 genes such as *PTGDR2*, *CD200R1*, and *KLRB1* (Fig. 1 C); however, the *IL7R*-low ILC2 cluster was enriched for the expression of type 2 cytokines such as *IL4*, *IL5*, and *IL13*, alarmins receptors such as *IL1RL1* (IL-33 receptor) and *IL17RB* (IL-25 receptor) as well as *PTGS2*, *DUSP4*, *BATF*, and *GATA3*, which suggested that this cluster holds an enhanced cell activation gene signature compared with *IL7R*-hi ILC2s (Fig. 1, A and D).

We also reanalyzed published scRNAseq data from skin punch biopsies of patients with AD (Alkon et al., 2022) (Fig. S1 C). This analysis revealed that the ILC2 clusters (0–3) can be further subdivided into two groups: one primarily characterized by the expression of genes typically associated with naive ILC2s, including *IL7R*, and the other marked by the expression of genes indicative of activated ILC2s, such as *IL5*, *IL13*, *IL1RL1*, and *IL17RB*, but with lower levels of *IL7R*. Consequently, *IL7R*-low ILC2s, which are enriched in cytokine and effector mRNA expression, were notably present in nasal polyp tissue and AD lesions (Fig. S1 D).

To confirm the presence of *IL7R*-low ILC2s in vivo, we performed further analysis of nasal polyp samples by flow cytometry. In our analyses, we identified a subset of CD127^−^ ILC2s, albeit present at a lower frequency than CD127^+^ ILC2s (Fig. 1 E). Furthermore, the expression of the surface marker CD45RO was higher in CD127^−^ ILC2s compared with CD127^+^ ILC2s, in agreement with their gene expression profile of cell activation (Fig. 1 F).

Given the negative correlation observed between *IL7R* expression and a gene signature of cell activation in ILC2s, we tested the responsiveness of CD127^−^ ILC2s. Purified CD127^−^ ILC2s from nasal polyps, obtained from CRSwNP patients with a blood eosinophil count of >250 cells/μl, showed an enhanced response to cytokine stimulation by producing higher amounts of type 2 cytokines compared with CD127^+^ ILC2s (Fig. 1 G). Of note, nasal polyps from patients with CRSwNP represent inflamed tissue (Fokkens et al., 2020), and therefore both CD127^+^ and CD127^−^ ILC2s are likely chronically activated.

Altogether, these data suggest that tissues with a type 2 inflammation signature, including nasal polyps and AD samples, contain CD127^−^ ILC2s characterized by an enhanced cell activation gene signature and greater ability to respond to stimuli.

### CD127^−^ ILC2s are present in healthy tissues

**Figure S2.**
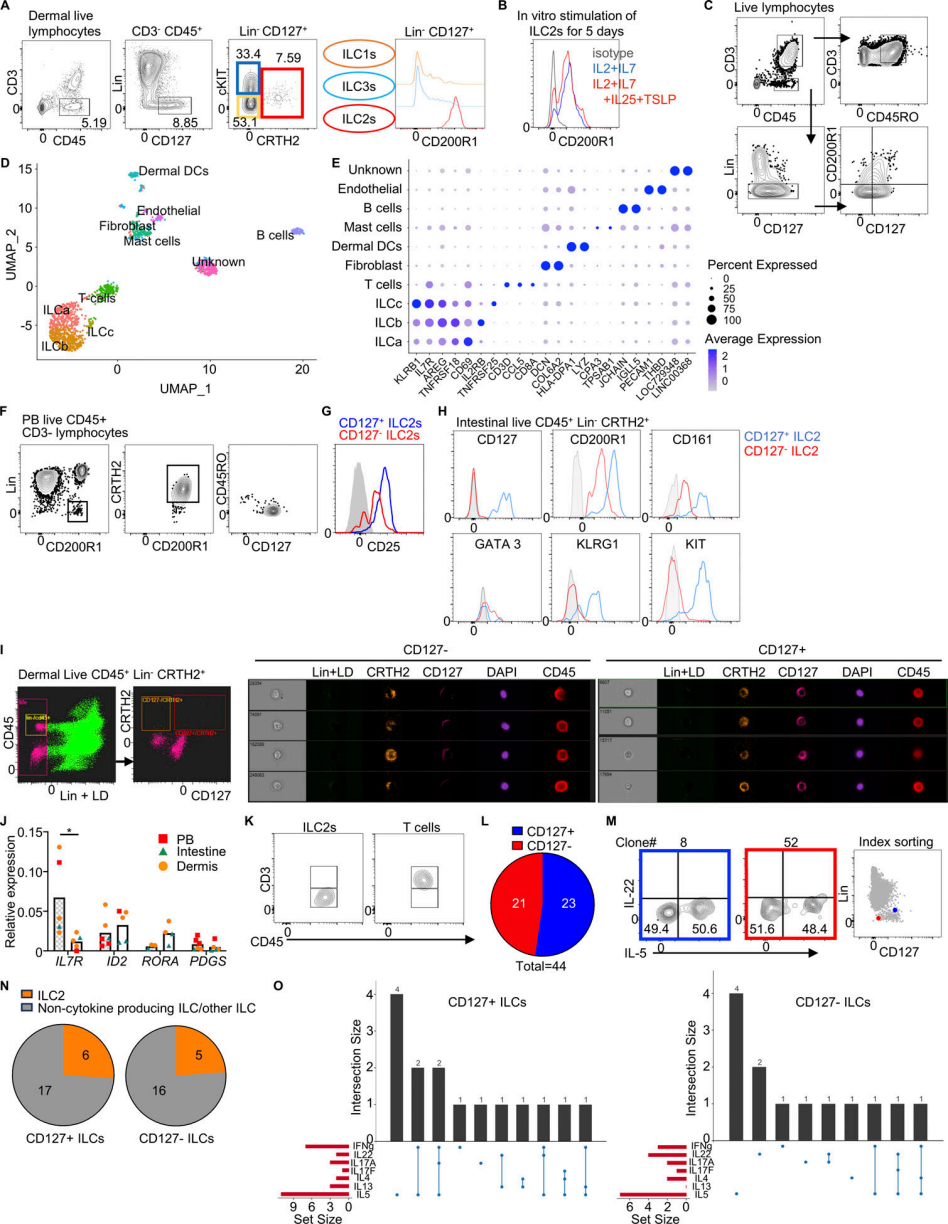
**CD127**^**−**^** ILC2s are present in healthy skin. (A)** A complete gating strategy and expression of CD200R1 of healthy human dermal ILCs analyzed by flow cytometry. **(B)** Flow cytometry measurement of CD200R1 expression on PB ILC2s after in vitro stimulation with IL-2+IL-7+IL-25+TSLP or IL-2+IL-7 as control for 7 days. **(C)** Gating strategy for sorting of human dermal Lin^−^ cells including ILCs for the scRNAseq experiment. Cells were gated on viable lymphocytes CD45^+^ Lin (CD1a, CD3, CD5, CD8, CD11c, CD14, CD16, CD19, CD34, CD94, CD123, FceR1a, TCRαβ, TCRγδ, BDCA2)^−^ and then gated in four quadrants using CD127 and CD200R1 expression. Equal numbers of the four populations were sorted. **(D)** UMAP visual representation of the cell clusters obtained from the scRNAseq analysis of Lin^−^ cells sorted from healthy dermis. Clusters were calculated using Seurat. DC, dendritic cells. **(E)** Dot plot representing the expression of marker genes from each cluster identified in the scRNAseq analysis. The intensity of the color indicates the level of expression of the indicated gene. The size of the symbol indicates the percentage of cells within the cluster expressing the corresponding marker genes. **(F)** Alternative gating strategy to identify CD127^+^ and CD127^−^ ILC2s in PB. **(G)** Histogram showing CD25 expression on CD127^+^ (blue) and CD127^−^ (red) ILC2s from PB. **(H)** Flow cytometry measurement of the expression of CD127, CD200R1, CD161, GATA3, KLRG1, and c-KIT on CD127^+^ and CD127^−^ ILC2s from adult intestine. **(I)** ImageStream analysis of CD127^+^ and CD127^−^ ILC2s from dermis. LD, live/dead staining. **(J)** qPCR of the expression of *IL7R*, *ID2*, *RORA*, and *PDGS* in CD127^+^ and CD127^−^ ILC2 isolated from PB, intestine, and dermis (*n* = 4–6). Pattern column = CD127^+^ ILC2s, empty column = CD127^−^ ILC2s. **(K)** ILC2s and T cells were purified by FACS from human blood and cultured in the presence of IL-2, IL-7, and IL-33 for 3 days. The absence of the expression of CD3 surface marker in cultured ILC2s is shown in the left panel. As a control for CD3^+^ cell gating, T cell staining pattern is shown on the right panel. **(L)** Pie chart showing the number of total clones from CD127^+^ and CD127^−^ ILCs obtained after single-cell sorting of ILCs from PB. **(M)** Flow cytometry measurement of IL-5 and IL-22 expression from ILC2 clones (left) and index sorting phenotype of the clones (right). **(N)** Pie charts showing the numbers of ILC2 and other ILC clones from CD127^+^ and CD127^−^ ILCs. **(O)** Summary of numbers and type of cytokines produced by CD127^+^ and CD127^−^ ILC clones. Data in A, B, G, H, and K are representative of at least three donors from at least three independent experiments. Symbols represent individual donors from at least three independent experiments; bars indicate mean values. *P ≤ 0.05 (paired *t* test).

**Figure 2.**
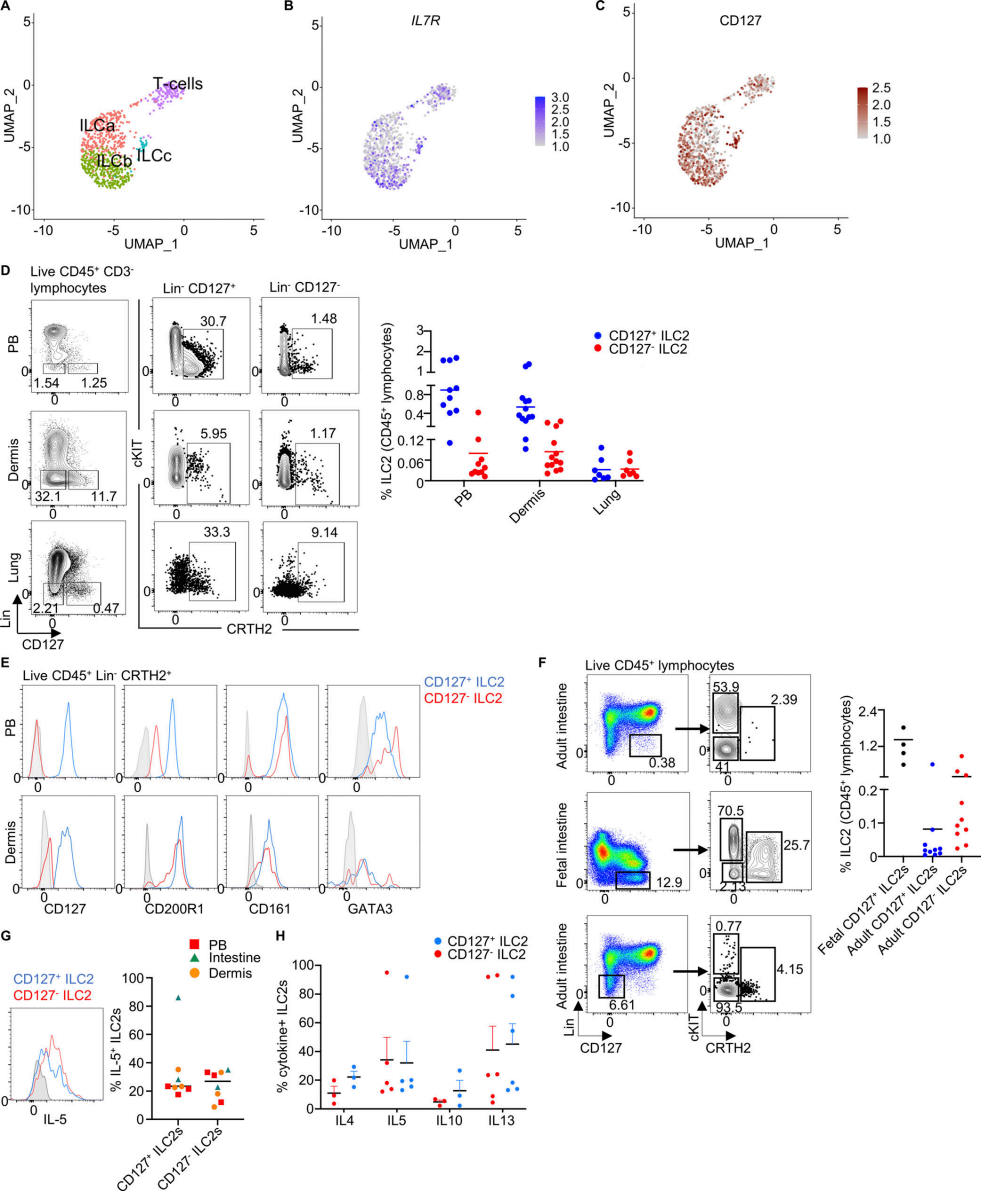
**CD127^−^ ILC2s are present in healthy tissues.** scRNAseq was performed in healthy human dermal cells (see gating strategy in Fig. S2 C). **(A)** UMAP visual representation of the ILC and T cell clusters obtained after filtering out non-relevant cell types in the Lineage-negative compartment. **(B)** Expression level of *IL7R* plotted in blue on the ILC and T cell clusters. The intensity of the color indicates the expression level. **(C)** Index sorting overlay of CD127-expressing cells on the UMAP clustering. Brown dots indicate CD127-expressing cells. The intensity of the color indicates the expression level. **(D)** Flow cytometry analysis and quantification of CD127^+^ and CD127^−^ ILC2s in fresh human PB, dermis, and lung (*n* = 7–13). Lin: CD1a, CD3, CD4, CD5, CD8, CD11b, CD11c, CD14, CD16, CD19, CD20, CD31, CD34, CD56, CD94, CD123, FcεR1α, TCRαβ, TCRγδ, and BDCA-2. **(E)** Flow cytometry measurement of the expression of CD127, CD200R1, CD161, and GATA3 on CD127^+^ and CD127^−^ ILC2s from PB and dermis. **(F)** Flow cytometry analysis and quantification of CD127^+^ and CD127^−^ ILC2s in adult and fetal intestine (*n* = 4–10). **(G)** CD127^+^ and CD127^−^ ILC2s were isolated from fresh PB, dermis, and adult intestine and in vitro stimulated with IL-2+IL-7+IL-33 (IL-25 for dermis)+TSLP for 5 days. Intracellular staining of IL-5 was performed and analyzed by flow cytometry (*n* = 8). **(H)** Quantification of the expression of the indicated cytokines in the CD127^+^ and CD127^−^ ILC2s isolated from PB and stimulated with IL-2+IL-7+IL-33+TSLP for 5 days (*n* = 3–6). scRNAseq data is representative of at least two independent experiments. scRNAseq data analysis was performed using Seurat. Data in E are representative of at least three donors from more than three independent experiments. Symbols represent individual donors from at least two independent experiments; bars indicate mean values ± SEM.

The observation of two distinct ILC2 cellular states in inflamed tissues led us to ask whether healthy tissues also contained CD127^−^ ILC2s. To this end, we purified CD45^+^Lin^−^ cells from healthy human dermis directly after surgical removal and analyzed them by scRNAseq. We performed plate-based scRNAseq (Muraro et al., 2016) to be able to back-trace protein expression by using index sorting at the time of the purification. The identification of a CD127^−^ ILC2 population called for another surface marker to identify ILC2s within Lin^−^ cells. CD200R1 has been shown to be expressed on all human and mouse ILC subsets in peripheral blood (PB), intestine, and tonsil but showed the highest expression on ILC2s (Nagasawa et al., 2019). We observed that dermal CD127^+^ ILC2s also highly expressed CD200R1, in contrast to ILC1 and ILC3s (Fig. S2 A), and CD200R1 expression on ILC2s did not change upon in vitro stimulation (Fig. S2 B). To enrich for ILCs within the Lin^−^ compartment and optimize our scRNAseq approach, equal numbers of cells were sorted into four distinct groups: CD127^+^CD200R1^+^, CD127^+^CD200R1^−^, CD127^−^CD200R1^+^, and CD127^−^CD200R1^−^ (Fig. S2 C). CD45^+^CD3^+^CD45RO^+^ T cells were added as a reference. From 1,583 cells sequenced, high-quality transcriptomes were obtained from 1,449 cells and further analyzed. Dimension reduction using uniform manifold approximation and projection (UMAP) revealed 10 clusters (Fig. S2 D), including ILCs and some non-ILC populations such as endothelial cells, fibroblasts, and dermal dendritic cells. ILCs, which expressed *KLRB1*, *IL7R*, *AREG*, *TNFRSF18*, and *CD69* and were negative for *CD3D*, were divided into three clusters: ILCa, ILCb, and ILCc (Fig. S2 E). Analysis of the distribution of *IL7R* expression within the ILC clusters showed that cluster ILCa expressed lower levels of *IL7R* mRNA compared with ILCb and ILCc (Fig. 2, A and B). This was corroborated by the level of CD127 protein expressed in these ILC populations (Fig. 2 C).

Flow cytometry analysis of several tissues including PB and dermis confirmed the presence of CD127^−^ ILC2s albeit at a lower frequency than CD127^+^ ILC2s (Fig. 2 D). Gating on Lin^−^ CD200R1^+^ cells first followed by a CRTH2^+^ gate confirmed the presence of CD127^−^ CD45RO^+^ ILC2s in PB (Fig. S2 F). These CD127^−^ ILC2s presented a typical ILC2 phenotype, expressing CD200R1, CD161, and GATA3 (Fig. 2 E). CD127^−^ ILC2s from PB also expressed CD25, while the expression levels were slightly lower in comparison with CD127^+^ ILC2s (Fig. S2 G). Remarkably, we also found ILC2s as CD127^−^ ILC2s in the adult intestine, where ILC2s are typically considered rare in humans despite their abundance in the fetal intestine (Fig. 2 F).

Imaging flow cytometry (ImageStream) analysis (Ortyn et al., 2006) confirmed the lymphocytic morphology of CD127^−^ ILC2s and showed low amounts of CD127 on the cell surface (Fig. S2 I). Quantitative PCR (qPCR) of *IL7R* showed that ILC2 from several tissues expressed lower levels of *IL7R* in CD127^−^ cells compared with CD127^+^ ILC2s, in agreement with the dermis scRNAseq data (Fig. S2 J), correlating mRNA and protein expression and therefore ruling out the possibility of technical artifacts introduced by tissue processing or immunophenotyping during our analyses. These data suggest that CD127^−^ ILC2s do not constitute a separate lineage independent of IL-7 signaling. Although these cells express low levels of *IL7R* transcripts, they appear negative for CD127 expression in flow cytometry analyses, and therefore we will be referring to them as CD127^−^ ILC2s.

To determine the functionality of CD127^−^ ILC2s, we purified Lin^−^CD127^−^CRTH2^+^ and Lin^−^CD127^+^CRTH2^+^ ILC2s from various tissues and stimulated them in vitro with an ILC2 activating cytokine cocktail. Upon 5 days of stimulation, Lin^−^CD127^−^CRTH2^+^ cells became positive for intracellular IL-5 staining (Fig. 2 G). Furthermore, other ILC2-related cytokines including IL-4, IL-10, and IL-13 were detected in PB CD127^− ^ILC2s (Fig. 2 H). To exclude any potential contamination from T or natural killer (NK) T cells in our in vitro cultures, the sorting strategy included gating out CD3^+^ cells before gating Lineage-negative cells. The lineage cocktail included antibodies against CD1a, CD3, CD4, CD5, CD8a, CD14, CD16, CD19, CD34, CD56, CD94, CD123, TCRαβ, TCRγδ, FcεR1α, and BDCA-2. For skin ILC2 purification, antibodies recognizing CD11b, CD11c CD20, and CD31 were additionally included in the lineage cocktail. Furthermore, when analyzing the purified ILC2s from in vitro cultures, we further stained for CD3 to allow us to gate out T cells excluding any potential contamination that may have occurred during sorting (Fig. S2 K).

To validate the identity of CD127^−^ ILC2s, we conducted single-cell cloning assays. We cultured Lin^−^ CD127^+^ and CD127^−^ from individual ILC2s, supported by mouse stromal cells (OP9-DL1), and in the presence of a cytokine cocktail consisting of IL-2, IL-7, IL1β, and IL-23, which is known to promote the expansion of all ILC subtypes (Krabbendam et al., 2021; Nagasawa et al., 2019). After 2 weeks, we analyzed the expanded clones (cloning efficiency of 14.2% and 12.2% for CD127^+^ and CD127^−^ cells, respectively) with 23 originating from the CD127^+^ ILCs and 21 from the CD127^−^ ILCs, for cytokine expression using flow cytometry (Fig. S2 L). 26% of the CD127^+^ and 23.8% of the CD127^−^ ILC clones were true ILC2s (producing IL-5, IL-4, IL-13, and/or IL-17F) (Fig. S2, M and N). Additionally, we identified ILC1 and ILC3 clones, as well as other clones with a plastic ILC phenotype (Fig. S2 O). Consequently, it is evident that the Lin^−^ CD127^−^ compartment indeed contains functional and bona fide ILC2s. Altogether, our data show that some functional ILC2s present in tissues and in the circulation of healthy individuals do not express CD127 on their cell surface.

### CD45RO expression is enriched within the CD127^−^ ILC2s in healthy tissues

**Figure 3.**
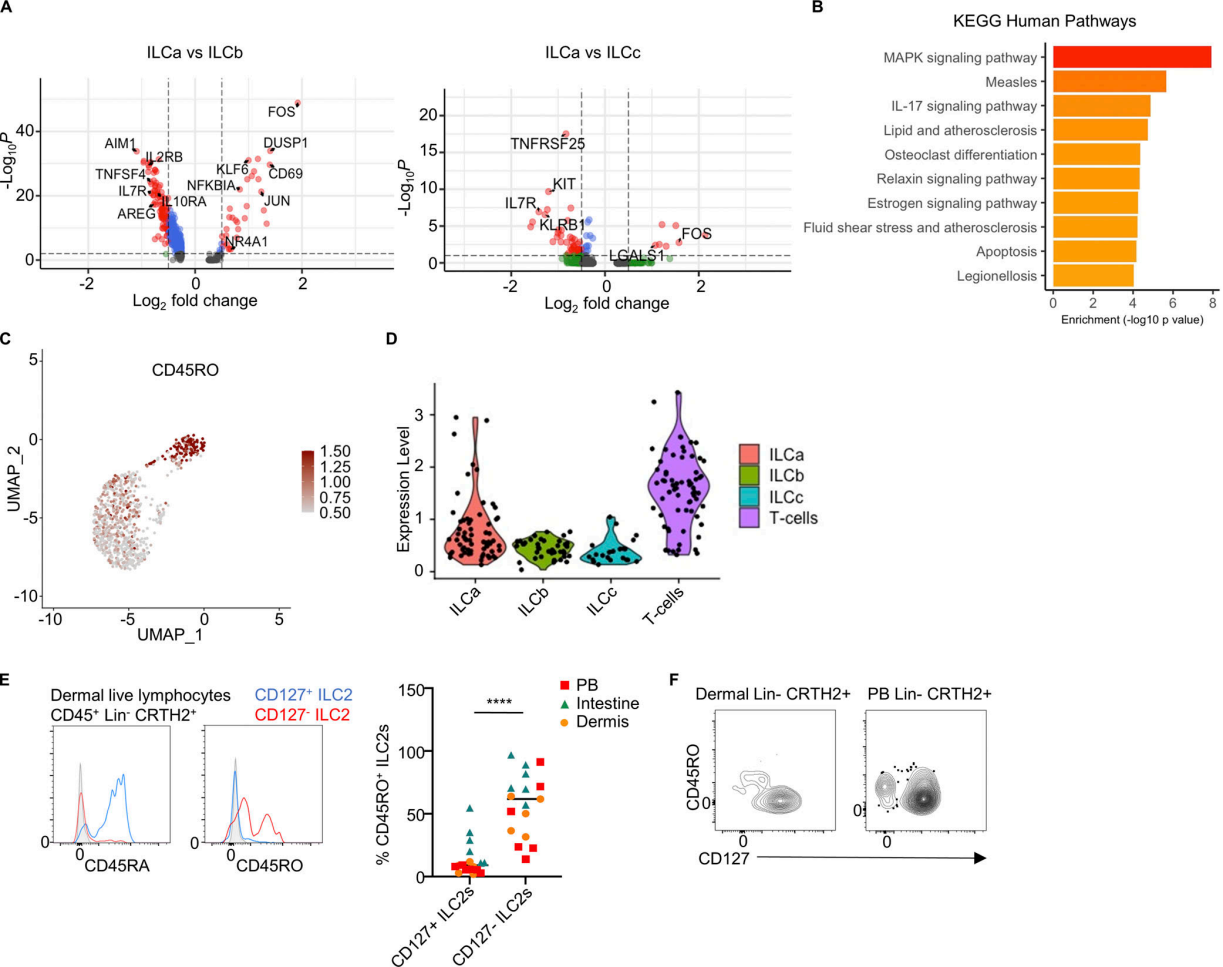
**CD127^−^ ILC2s express high levels of CD45RO in healthy tissues. (A)** Volcano plots showing differential gene expression between the ILC clusters. Red color marks genes above the P value and fold change thresholds (P < 0.001, log fold change > 0.5). Statistical significance was calculated using a non-parametric Wilcoxon rank sum test. **(B)** Pathway enrichment based on differential gene expression for cluster ILCa compared with ILCb. KEGG, Kyoto Encyclopedia of Genes and Genomes. **(C)** Index sorting overlay of CD45RO expressing cells on the UMAP reclustering. Red dots indicate CD45RO-expressing cells. **(D)** Expression levels of CD45RO protein in CD200R1^+^ cells in each cluster. **(E)** Flow cytometry measurement of CD45RA and CD45RO expression on CD127^+^ and CD127^−^ ILC2s from dermis (histograms) and quantification of the percentage of CD45RO expression on CD127^+^ and CD127^−^ ILC2s from the indicated tissues (*n* = 17). Symbols represent individual donors from at least three independent experiments; bars indicate mean values. **(F)** The contour plots show the expression of CD45RO and CD127 in human dermis (left) and PB (right) ILC2s. ****P ≤ 0.0001 (paired *t* test). scRNAseq data analysis was performed using Seurat. Data in F are representative of at least three donors from three independent experiments.

**Figure S3.**
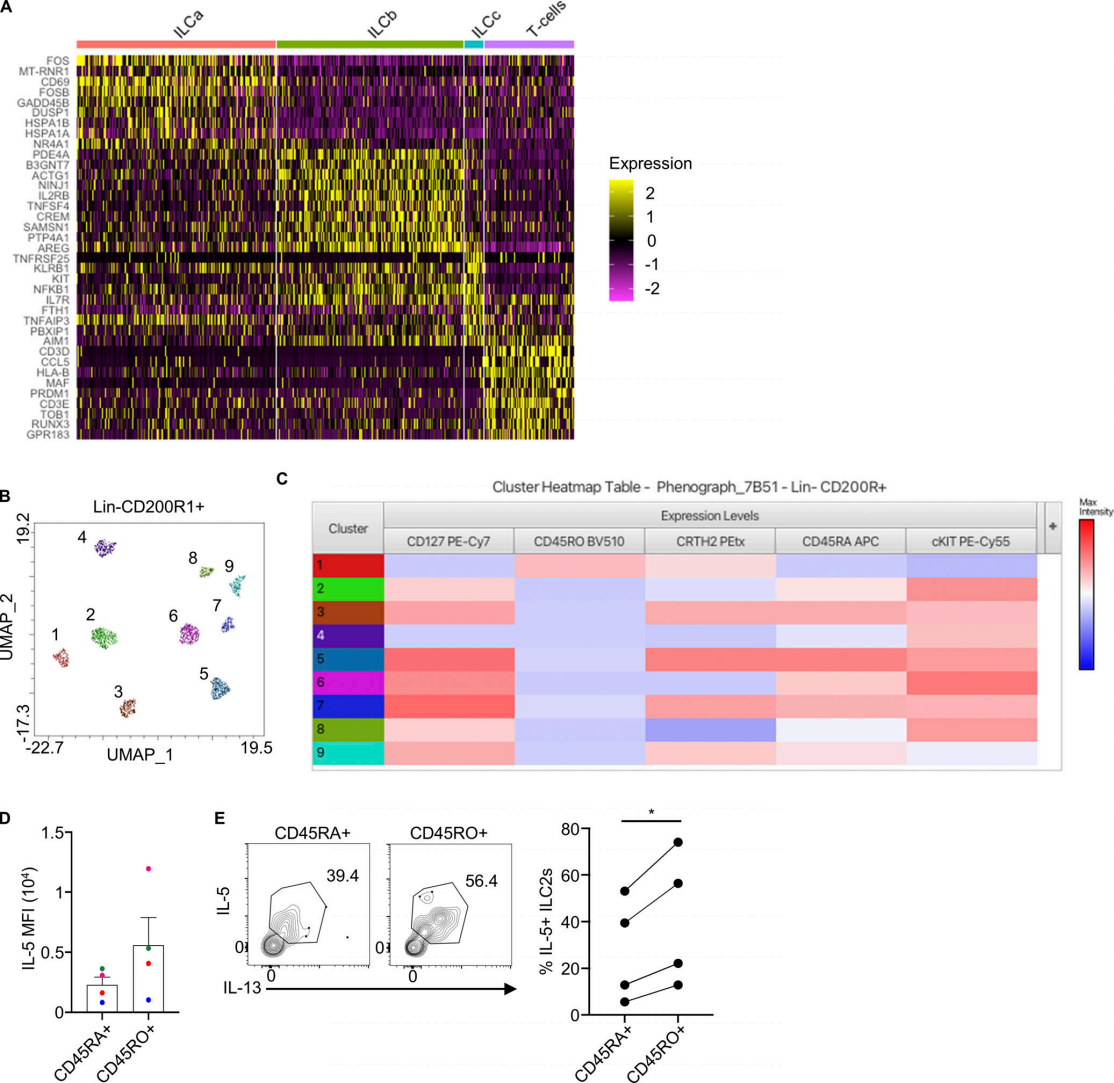
**CD127**^**−**^**CD45RO^+^ ILC2s are present in healthy tissues and show enhanced responsiveness. (A)** Heatmap showing expression of top marker genes for each ILC and T cell cluster. **(B)** Unbiased clustering of Lineage^−^ CD200R1^+^ cells from human dermis visualized using UMAP projection. **(C)** Heatmap showing relative expression of the designated markers in each cluster. Expression levels are shown in colors following the legend on the right. Clustering analysis was performed using the Phenograph tool in FlowJo v10.0.0. Data are representative of at least two independent experiments. **(D)** MFI for IL-5 of CD45RA^+^ and CD45RO^+^ ILC2s isolated from PB and in vitro stimulated with IL-2+IL-7+IL-33+TSLP for 24 h (*n* = 4). **(E)** Purified blood ILC2s were stimulated with PMA (50 ng/ml) + ionomycin (500 nM) + IL-2 (20 ng/ml) for 24 h. During the last 3 h, GolgiStop was added to the culture. FACS plots show intracellular staining of IL-5 in CD45RA^+^ and CD45RO^+^ ILC2s. The graph on the right shows the percentages of IL-5^+^ ILC2s (*n* = 4). Symbols represent individual donors from at least two independent experiments. *P ≤ 0.05 (paired *t* test)

To determine differentially expressed genes between the ILC clusters in the dermis, we performed a non-parametric Wilcoxon rank sum test. In addition to the significantly lower expression of *IL7R* in ILCa compared with ILCb and ILCc, this cluster was characterized by the expression of activation-related genes including *CD69*, *DUSP1*, *FOS*, *JUN*, *KLF6*, *NFKBIA*, and *NR4A1* (Fig. 3 A and Fig. S3 A). Accordingly, pathway enrichment analysis showed a gene signature for MAPK activation as the top pathway represented in the ILCa cluster (Fig. 3 B). Our analyses showed that skin ILCs are more easily discriminated based on the cellular state of activation rather than predefined ILC identity. This is consistent with previous reports describing ILC infidelity (i.e., co-expression of marker genes typically associated with ILC2s and ILC3s) in mouse and human skin (Bielecki et al., 2021; Alkon et al., 2022). Index sorting data showed enrichment of CD45RO^+^ cells within the ILCa cluster (Fig. 3, C and D), consistent with our previous analyses in nasal polyps. Flow cytometry analysis confirmed that CD127^−^ ILC2s expressed CD45RO and were negative for CD45RA, in contrast to CD127^+^ ILC2s (Fig. 3 E and Fig. S3, B and C). Importantly, the majority of CD45RO^+^ ILC2s were negative for CD127 expression, except for PB where CD127^+^CD45RO^+^ ILC2s were also detected, suggesting that modulation of CD127 may depend on tissue signals (Fig. 3 F). Therefore, CD127^−^CD45RO^+^ ILC2s are not only present in inflamed tissues but also in healthy tissues, carrying a gene signature of cell activation.

### CD127^−^CD45RO^+^ ILC2s from healthy donors are more responsive than CD127^+^CD45RA^+^ ILC2s

**Figure 4.**
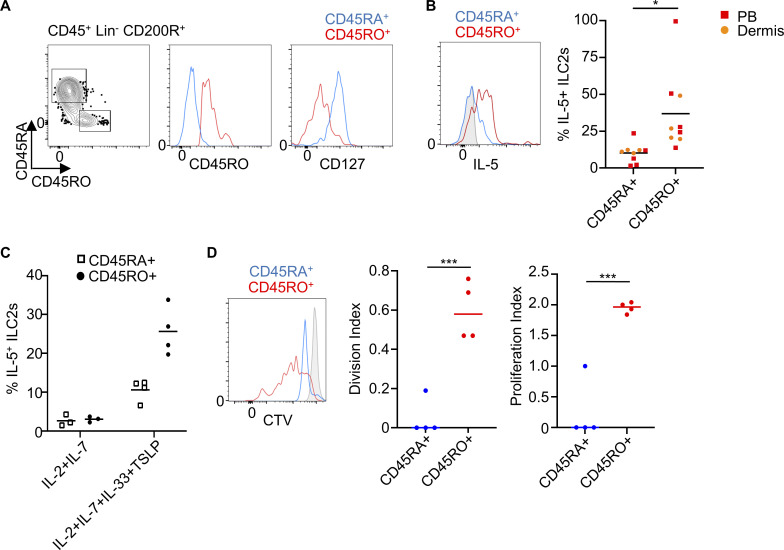
**CD45RO^+^ ILC2s from healthy donors are more responsive than CD45RA^+^ ILC2s. (A)** Sorting strategy of CD45RA^+^ (CD127^+^) and CD45RO^+^ (CD127^−^) ILC2s isolated from healthy human dermis (contour plot). The histograms show the expression of CD45RO and CD127 in sorted CD45RO^+^ and CD45RA^+^ ILC2s. An equal number of cells were plated in a round-bottom 96-well plate. **(B)** Histogram and quantification showing IL-5 expression on stimulated CD45RA^+^ and CD45RO^+^ ILC2s from the indicated tissues (*n* = 9). PB ILC2s were stimulated with IL-2+IL-7+IL-33+TSLP and dermal ILC2s with IL-2+IL-7+IL-25+TSLP for 24 h. **(C)** Percentage of IL-5 expression on CD45RA^+^ and CD45RO^+^ dermal ILC2s after 24 h stimulation with IL2+IL7+IL33+TSLP or IL2+IL7 as control (*n* = 3–4). **(D)** CTV staining of in vitro–stimulated CD45RA^+^ and CD45RO^+^ ILC2s purified from healthy dermis as in A, with the same cytokine cocktail as in B for 48 h. (*n* = 4). Symbols represent individual donors from at least two independent experiments; bars indicate mean values. *P ≤ 0.05 and ***P ≤ 0.001 (paired *t* test).

We have previously observed that mouse memory ILC2s are characterized by increased transcription of genes related to ILC2 activation (Martinez-Gonzalez et al., 2016). Therefore, we hypothesized that human CD127^−^CD45RO^+^ ILC2s in healthy tissues, marked by an activation gene signature, could have been previously activated ILC2s that remained in the tissue. In fact, it is conceivable that healthy tissues, especially surface barriers, contain previously activated or experienced ILC2s as our body is constantly exposed to extrinsic stimuli that can activate ILC2s. In agreement, previously activated tissue-resident memory T cells are marked by the expression of CD45RO (Bunce and Bell, 1997). To investigate the potential immunological memory properties of CD127^−^CD45RO^+^ ILC2s, we purified CD127^−^CD45RO^+^ and CD127^+^CD45RA^+^ ILC2s from healthy PB and dermis by FACS (Fig. 4 A). To study the rapid response typically found in immune memory cells, we performed a 24 h in vitro stimulation with an ILC2 stimulatory cytokine cocktail (IL-2+IL-7+IL-33+TSLP and IL-2+IL-7+IL-25+TSLP for PB and dermis, respectively). CD45RO^+^ ILC2s produced significantly larger amounts of IL-5 than CD45RA^+^ ILC2s (Fig. 4 B and Fig. S3 D). This observation was also true when using non-specific (PMA and ionomycin) stimulation for 24 h (Fig. S3 E). CD45RO^+^ ILC2s did not produce cytokines when cultured with IL-2+IL-7 (Fig. 4 C), suggesting that these cells were not actively producing cytokines in the tissue. Finally, CD45RO^+^ ILC2s from dermis showed increased proliferation in response to a 48 h stimulation with IL-2+IL-7+IL-25+TSLP compared with CD45RA^+^ ILC2s (Fig. 4 D). As mentioned earlier, CD127^−^ ILC2s express low levels of CD127 on the cell surface, which might enable TSLP to activate them.

In summary, these findings indicate that CD127^−^CD45RO^+^ ILC2s are present in both the bloodstream and as resident cells within tissues. Moreover, they exhibit a more rapid and robust cytokine production capacity when compared with CD127^+^CD45RA^+^ ILC2s, strongly implying their identity as memory ILC2s.

### CD127^−^ ILC2s are present in mouse tissues

**Figure 5.**
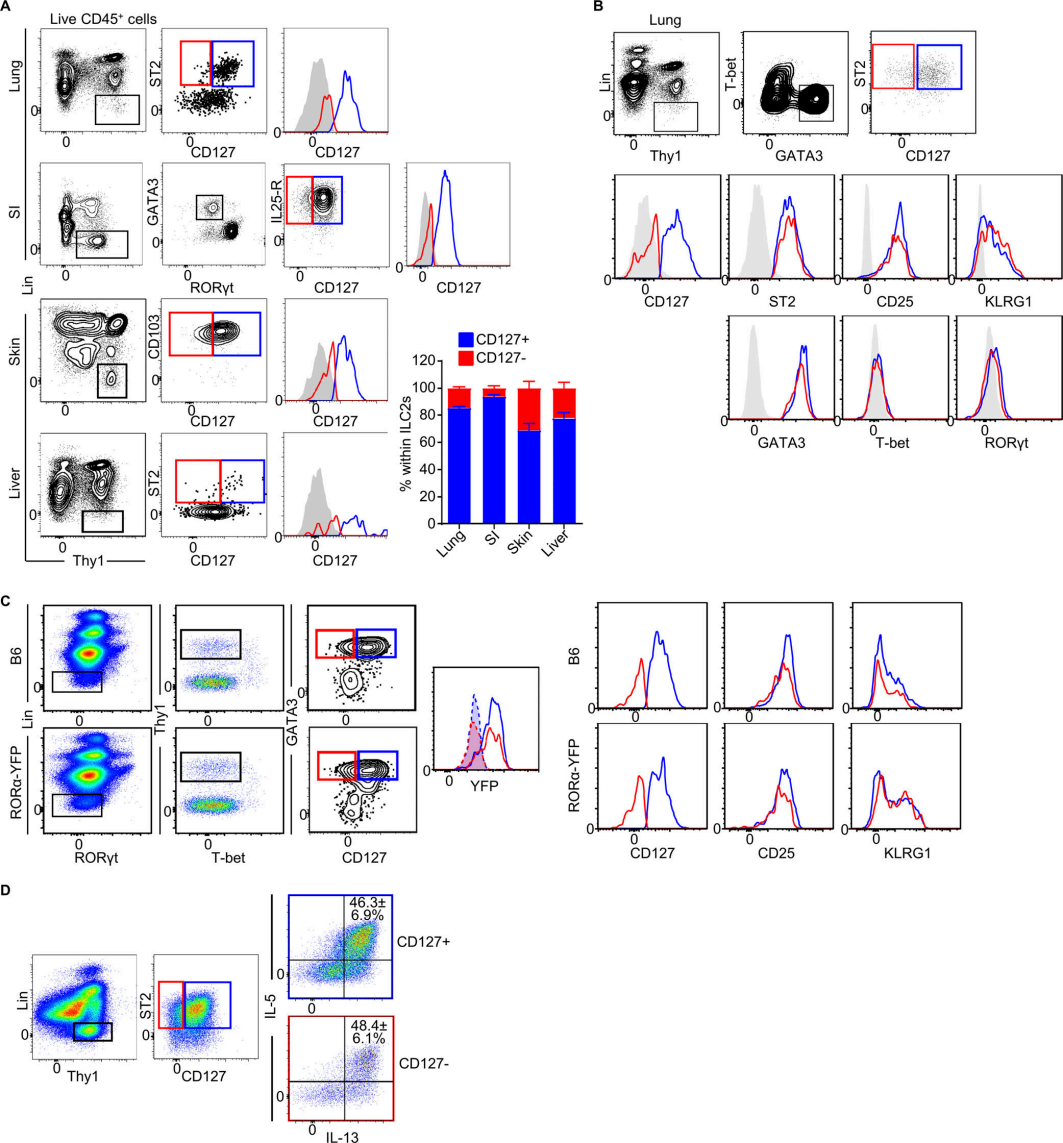
**CD127^−^ ILC2s are present in mouse tissues. (A)** Gating strategies used to quantify CD127^+^ (blue) and CD127^−^ (red) ILC2s in various tissues (plots on the left) and quantification of the two subsets (graph on the right). The histograms show the expression levels of CD127 on CD127^+^ (blue) and CD127^−^ (red) ILC2s, together with a lineage-high population negative for CD127 (gray). **(B)** Phenotypic characterization of CD127^+^ (blue) and CD127^−^ (red) ILC2 in mouse lungs. **(C)** Identification and phenotypic characterization of CD127^+^ (blue) and CD127^−^ (red) ILC2s in B6 and RORα-YFP mouse lungs. **(D)** IL-5 and IL-13 expressions in CD127^+^ and CD127^−^ ILC2 subsets from B6 mouse lungs after 3 consecutive days of IL-33 stimulation. Percentages show the frequency of cells within IL-5^+^IL-13^+^ quadrant. Data presented are mean ± SEM. For skin data, *n* = 4, one experiment; for small intestine (SI), *n* = 6, two independent experiments; for lung, *n* = 13, four independent experiments; for liver, *n* = 11, four independent experiments (A); *n* = 6 (B and C); *n* = 7, three independent experiments (D).

**Figure S4.**
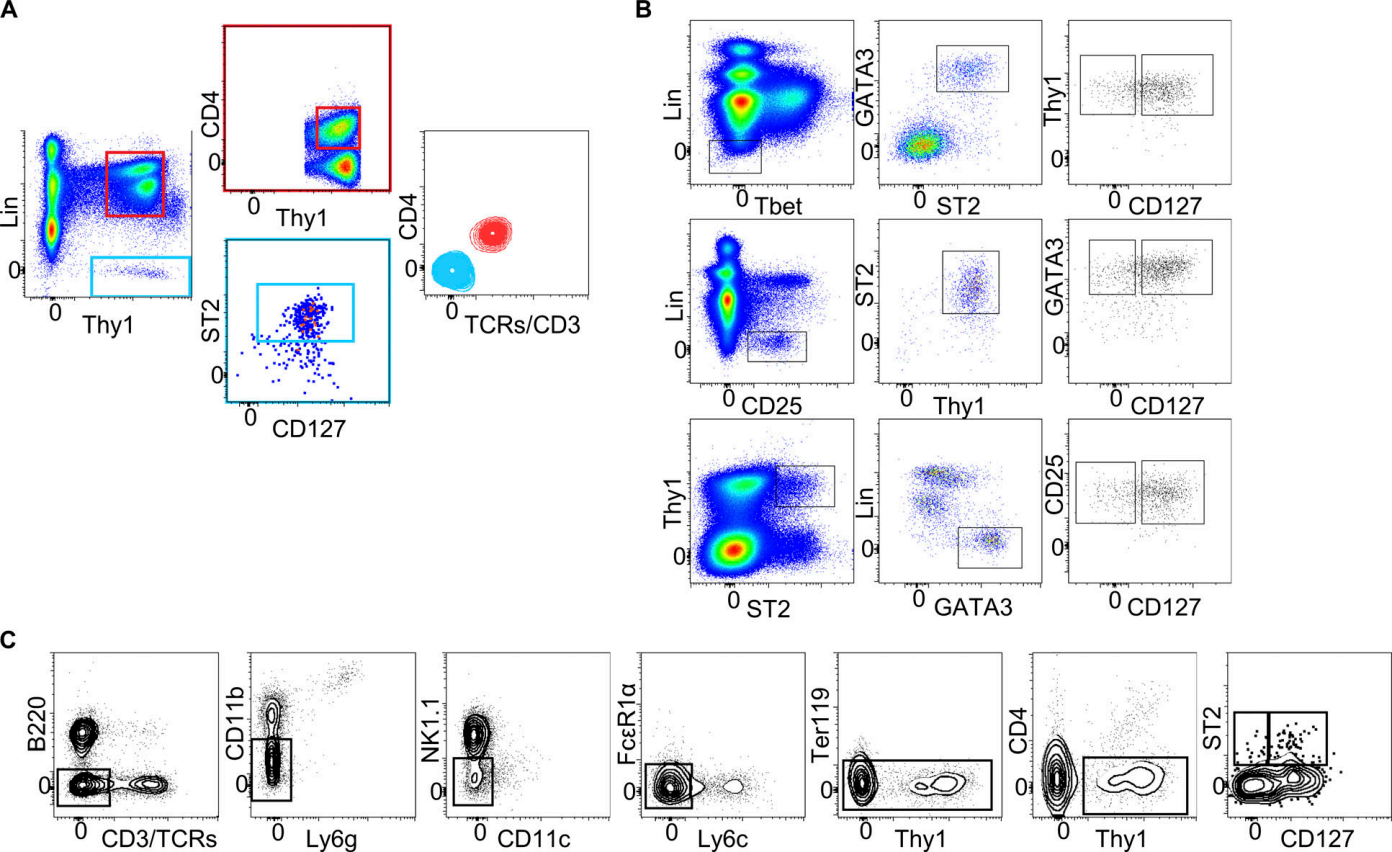
**Mouse CD127^−^ ILC2s can be found using different gating strategies. (A)** Gating of the mouse lung cells showing that there is no T cell contamination in our ILC2 gate. **(B)** Alternative gating strategy to identify CD127^+^ and CD127^−^ ILC2s in mouse lung. **(C)** Alternative gating strategy to identify CD127^+^ and CD127^−^ ILC2s, using individual lineage antibodies conjugated to different fluorochromes. FACS plots are representative of at least three independent experiments.

To investigate whether CD127^−^ ILC2s are present in mouse tissues, we analyzed naive B6 lungs, small intestine, skin, and liver by flow cytometry and identified CD127^+^ and CD127^−^ populations within respective ILC2 gates (Fig. 5 A). Fig. S4 shows that there is no T cell contamination in our ILC2 gate (Fig. S4 A) and that CD127^+^ and CD127^−^ ILC2s can be found using alternative gating strategies (Fig. S4 B), including when individual lineage antibodies were conjugated to different fluorochromes (Fig. S4 C). In the lung, CD127^+^ and CD127^−^ ILC2 populations expressed similar levels of ILC2-related surface markers and transcription factors including ST2, CD25, KLRG1, and GATA3, but not T-bet and RORγt, demonstrating phenotypic similarities between CD127^−^ ILC2s and CD127^+^ ILC2s (Fig. 5 B). To further confirm an ILC2 lineage identity, we analyzed the RORα-lineage tracer RORα-YFP mice, in which almost 90% of ILC2s express YFP (Ghaedi et al., 2020). CD127^+^ and CD127^−^ subsets of ILC2s defined by Lin^−^RORγt^−^T-bet^−^Thy1^+^GATA3^+^ cells expressed YFP and ILC2-related surface markers, confirming that they belong to the RORα^+^ ILC2 lineage (Fig. 5 C). Intracellular staining of the lungs after IL-33 treatment demonstrated that CD127^+^ and CD127^−^ ILC2s have a similar ability to produce IL-5 and IL-13, indicating that the CD127^−^ cells are functional ILC2s (Fig. 5 D). In this experimental setting, we cannot conclude whether naive CD127^−^ ILC2s were more responsive than CD127^+^ ILC2s since these mice received three consecutive i.n. administrations of IL-33, which constitutes a very potent stimulus that may saturate ILC2 responses. Altogether, these results demonstrate that CD127^−^ ILC2s in mice phenotypically and functionally resemble conventional CD127^+^ ILC2s, suggesting that they belong to the ILC2 lineage. Furthermore, their existence in mice supports the identification of the previously unappreciated CD127^−^ population in humans.

### Memory ILC2s in mice present reduced expression of CD127

**Figure 6.**
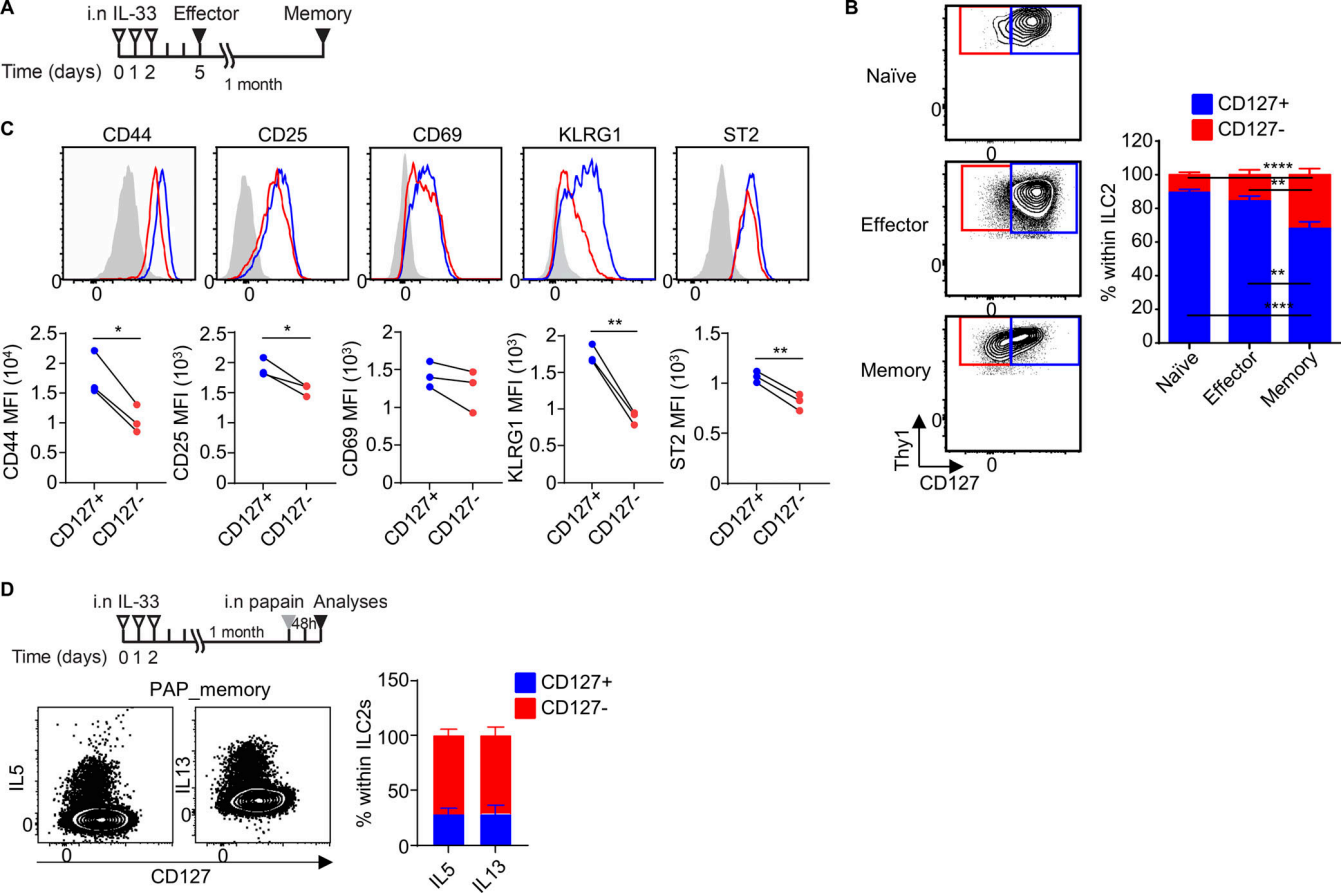
**Memory ILC2s in mice present reduced expression of CD127. (A)** Mice were treated with 3 consecutive days of i.n. IL-33 injections and naive, effector (72 h after the last injections), and memory (1 mo after the last injections) lungs were harvested. **(B)** CD127^+^ (blue) and CD127^−^ ILC2s (red) were analyzed by flow cytometry in naive, effector, and memory cells. **(C)** Memory lung CD127^+^ and CD127^−^ ILC2s were analyzed for the expression of CD44, CD25, CD69, KLRG1, and ST2, and the MFI of the expression levels was quantified. **(D)** Mice received 3 consecutive days of i.n. IL33 treatment and were challenged with one dose of i.n. papain 1 mo later. The lungs were harvested 48 h after the challenge. The dot plots show IL-5 and IL-13–producing ILC2s, while the graph shows the percentages of IL-5^+^ and IL-13^+^ ILC2s in the CD127^+^ (blue) and CD127^−^ (red) compartments. Data presented are mean ± SEM. *N* = 6, two independent experiments (B); *n* = 3, representative of at least two independent experiments (C and D). Two-way ANOVA (B) or paired two-tailed *t* test (C) was used to determine the statistical significance, with a P value ≤ 0.05 being significant. *P ≤ 0.05, **P ≤ 0.01, and ****P ≤ 0.0001.

We have previously shown that memory ILC2s, generated in a mouse model of allergic lung inflammation, undergo intrinsic changes that provide them with an increased reactivity to subsequent unrelated challenges (Martinez-Gonzalez et al., 2016). To investigate whether memory ILC2s in mice also modulated the expression of CD127, we analyzed ILC2s in the lungs of naive, effector (72 h after IL-33 i.n. administration), and memory (1 month after IL-33 i.n. administration) mice (Fig. 6 A). The percentage of CD127^−^ ILC2s was similar in naive and effector lungs, while it was significantly increased in memory mouse lungs (Fig. 6 B). Thus, the remodeling of the allergen-experienced ILC2 population was reflected in a larger CD127^−^ compartment. Marker expression analysis among CD127^+^ and CD127^−^ ILC2s in memory lungs showed similar expressions of CD69, while the expression levels of CD44, CD25, KLRG1, and ST2 were slightly lower in CD127^−^ ILC2s compared with CD127^+^ ILC2s, suggesting that they are phenotypically different (Fig. 6 C). Upon a single challenge with papain to generate an ILC2 memory response, most of the cytokines were derived from the memory CD127^−^ ILC2s rather than CD127^+^ ILC2s (Fig. 6 D). Of note, ILC2s expressing high levels of CD127 did not produce any cytokines. As observed previously (Martinez-Gonzalez et al., 2016), unprimed ILC2s did not response to a single challenge of papain. These findings indicate that memory ILC2s in mice are enriched within the CD127^−^ subset, mirroring our observations with human ILC2s.

### In vitro stimulation of CD127^+^ ILC2s generated highly responsive CD127^−^ CD45RO^+^ ILC2s

**Figure S5.**
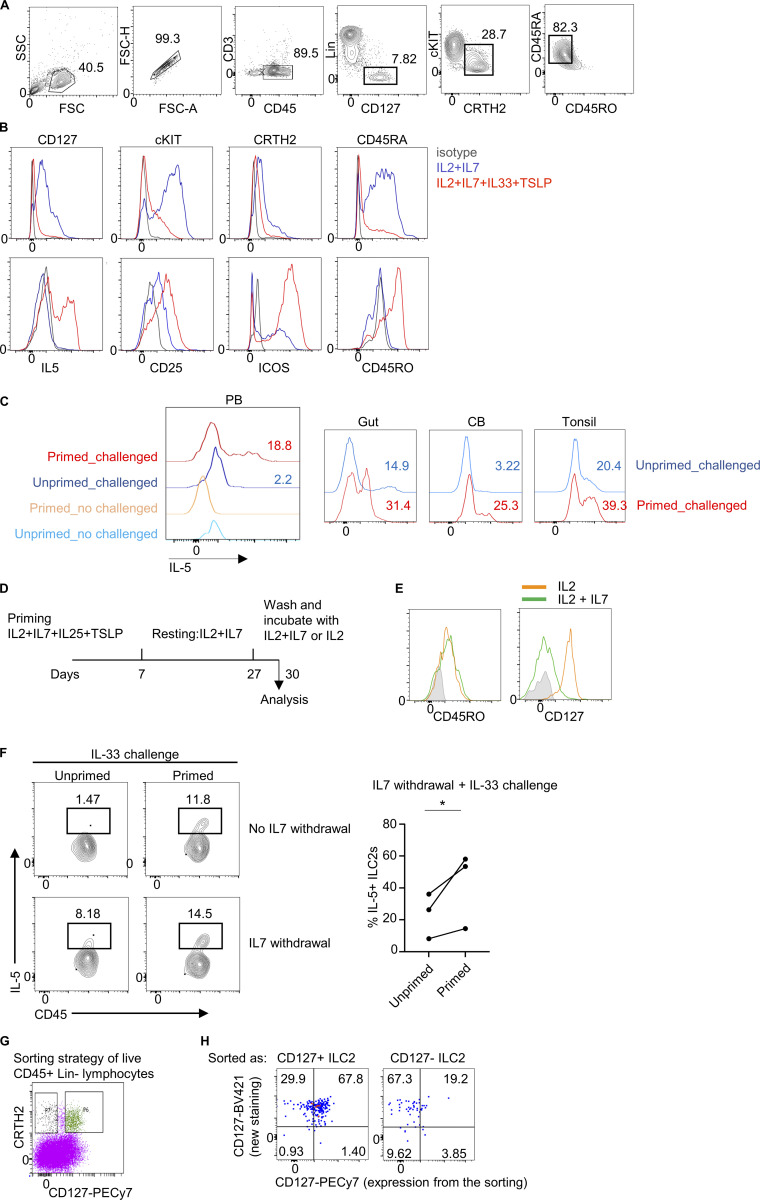
**Memory ILC2s modulate CD127 expression and do not require IL-7 for memory responses. (A)** Gating strategy for ILC2 sorting from PB (as an example) for the long-term in vitro culture experiment. **(B)** Flow cytometry measurements of the expression of the indicated markers on in vitro–stimulated (red) and control (blue) ILC2s on day 7. **(C)** Flow cytometry measurement of IL-5 expression of challenged (primed or unprimed) ILC2s isolated from the indicated tissues. CB, cord blood. **(D)** Scheme of the long-term in vitro culture with IL-7 withdrawal. **(E)** Histogram of the expression of CD45RO and CD127 on the cultured ILC2s in the indicated conditions. **(F)** Sorted CD45RA^+^ ILC2s from PB were primed (IL-2+IL-7+IL-33+TSLP) or unprimed (IL-2 + IL-7) for 7 days. Media was washed and cells were maintained on media supplemented with IL-2+IL-7 for 1 mo (media was refreshed every week). IL-7 was withdrawn from the culture (bottom plots) and 3 days later all wells were challenged with IL-33 for 24 h. Plots show IL-5% in the different conditions and the graph shows the quantification of IL-5+ ILC2s in unprimed and primed conditions after IL-7 withdrawal. Symbols represent individual donors (*n* = 3) from three independent experiments. *P ≤ 0.05 (paired *t* test). **(G)** Sorting strategy of CD127^+^ and CD127^−^ ILC2s from healthy dermis for the short-term in vitro culture in the presence of IL-2. **(H)** Flow cytometry analysis of CD127 expression on CD127^+^ and CD127^−^ isolated dermal ILC2s and cultured in the presence of IL-2 for 24 h. CD127 was stained with an antibody labeled with a different fluorochrome but the same clone as the one used for the sorting. Data in A–C, E, G, and H are representative of at least three donors from three independent experiments.

**Figure 7.**
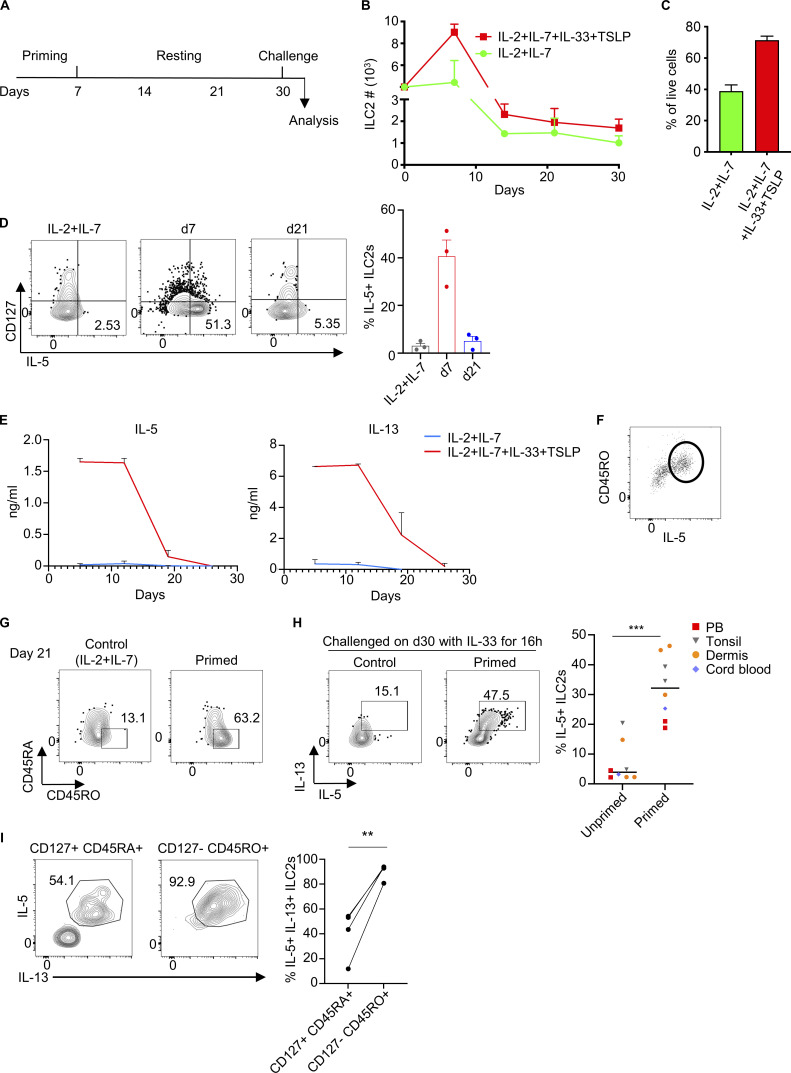
**In vitro–generated human CD45RO^+^ ILC2s showed immunological memory properties. (A)** Scheme of in vitro priming and challenge of purified CD127^+^CD45RA^+^ ILC2s isolated from healthy PB or dermis. ILC2s were either primed with IL-2+IL-7+IL-33+TSLP or maintained in IL-2+IL-7 as control for 7 days. Then, wells were washed, and media was refreshed with IL-2+IL-7 every week. ILC2s were challenged with IL33 or PBS as control on day 30 and were stained and analyzed by flow cytometry on day 31 after PMA+ionomycin+GolgiStop incubation for 3 h. **(B)** Time course analysis of the numbers of ILC2s during the in vitro culture experiment as in A. **(C)** Percent viable cells in the cultured ILC2s at different time points. **(D)** Flow cytometry measurement of CD127 and IL-5 expression on cultured ILC2s (contour plots) and quantification of IL-5^+^ ILC2s (graph) at indicated time points of the culture. **(E)** Time course of IL-5 and IL-13 levels in the supernatant of the cultured ILC2s quantified by ELISA. **(F)** Expression of CD45RO and IL-5 of primed ILC2s on day 7. **(G)** Flow cytometry measurement of CD45RA and CD45RO of cultured ILC2s on day 21. **(H)** Flow cytometry measurement and quantification of IL-5 expression on unprimed (IL-2+IL-7) and primed (IL-2+IL-7+IL-33+TSLP) ILC2s receiving IL-33 challenge (*n* = 8). **(I)** CD127^+^CD45RA^+^ and CD127^−^CD45RO^+^ were purified from PB and stimulated in vitro in the absence of IL-7, with only IL-2 and IL-33 for 24 h. FACS plots show the percentage of IL-5 and IL-13 in these ILC2s (left) and the graph shows the quantification (right) (*n* = 3). *N* = 3 in B–E from at least two independent experiments. Data shown in F and G are representative of at least three donors from at least two independent experiments. Symbols represent individual donors from at least three independent experiments; bars indicate mean values ± SEM. **P ≤ 0.01 and ***P ≤ 0.001 (paired *t* test). d, day.

We have observed that stimulation of human ILC2s induces downregulation of CD127 expression. To investigate whether CD127^+^ ILC2s could give rise to memory CD127^−^ CD45RO^+^ ILC2s, we sorted CD127^+^CD45RA^+^ ILC2s from several tissues including dermis, PB, intestine, tonsil, and cord blood (see a complete gating strategy from PB as an example in Fig. S5 A) and in vitro stimulated them with IL-2+IL-7+IL-33+TSLP for 7 days. The combination of IL-2 and IL-7 was used to maintain ILC2s alive as a control (Fig. 7 A). After 1 week of stimulation, the cells were washed and fresh media containing only IL-2+IL-7 were added to induce a resting state. Under these conditions, we observed that human ILC2s expanded in numbers upon activation, followed by a contraction phase upon alarmin withdrawal, during which a small ILC2 population survived and remained alive for up to 1 month (Fig. 7 B). Primed ILC2s showed an increased survival than control ILC2s at day 21 (71.5 ± 3.6 versus 38.9 ± 5.5 % of viable cells, respectively) (Fig. 7 C). Following stimulation, ILC2s underwent a phenotypic change characterized by the increased expression of CD45RO and the reduced expression of CD127 (Fig. S5 B). Throughout the expansion phase, ILC2s continued to release IL-5 and IL-13 (Fig. 7, D and E). In line with previous observations, cytokines were mostly produced by CD127^−^CD45RO^+^ ILC2s (Fig. 7 F). ILC2s retained the expression of CD45RO during the culture’s resting phase even when they were no longer actively producing cytokines (Fig. 7 G). To determine whether these previously activated ILC2s were more responsive to a secondary challenge compared with unprimed cells, we added IL-33 to the culture for a brief period (16 h) on day 30 and analyzed cytokine production by flow cytometry. Primed ILC2s strongly responded to the IL-33 challenge by producing IL-5 and IL-13, in contrast to a negligible response observed in unprimed ILC2s (maintained with only IL-2+IL-7 for 30 days) (Fig. 7 H and Fig. S5 C).

Interestingly, in vitro–generated CD127^−^CD45RO^+^ ILC2s regained CD127 expression when IL-7 was withdrawn from the culture (Fig. S5 D–F), suggesting that CD127 expression can be regulated. Nevertheless, they still maintained the expression of CD45RO and memory capacities. This observation prompted us to examine whether CD127^−^ ILC2s isolated from healthy tissues could regain the expression of CD127. We isolated dermal CD127^−^ ILC2s (and CD127^+^ ILC2s as control) and cultured them for 24 h in the presence of IL-2 (Fig. S5 G). The majority of the sorted CD127^−^ ILC2s upregulated the surface expression of CD127 (Fig. S5 H). Of note, we used two different fluorescent labels for the CD127 antibody at the time of the sorting and after the overnight culture. Then, we purified CD127^+^CD45RA^+^ and CD127^−^CD45RO^+^ from healthy PB and stimulated them with IL-2 and IL-33 and without IL-7 for 24 h. CD127^−^CD45RO^+^ ILC2s showed an enhanced intracellular expression of IL-5 and IL-13 by FACS compared with CD127^+^CD45RA^+^ ILC2s, indicating that IL-7 was not required for memory responses (Fig. 7 I).

In conclusion, human CD45RA^+^ ILC2s become CD127^−^CD45RO^+^ ILC2s upon stimulation. Once they return to a resting state, they retain CD45RO expression and are intrinsically more responsive to restimulation compared with unprimed ILC2s, suggesting they have acquired immunological memory. Unlike CD45RO, the expression of CD127 in memory ILC2s seems to be adaptable to external signals.

## Discussion

In this study, we define human memory ILC2s as resting Lin^−^CRTH2^+^CD200R1^+^CD127^−^CD45RO^+^ cells that remember past activation events, leading to a more pronounced reaction to subsequent stimuli compared with their initial response. In comparison with CD127^+^CD45RA^+^ ILC2, they exhibit increased proliferation rates and produce larger quantities of type 2 cytokines when exposed to epithelial alarmins. Expression of CD45RO in ILC2s found in healthy tissues indicates that they have previously undergone activation events. It is worth noting that memory ILC2s may also exist within the CD127^+^ CD45RO^+^ ILC2 subset, as observed in nasal polyps from patients with CRSwNP, suggesting that downregulation of CD127 is a dynamic process in the development of memory ILC2s. However, in healthy tissues like the skin, CD127^+^ ILC2s predominantly express CD45RA.

ILCs rely on IL-7 for their development (Sheikh and Abraham, 2019) and therefore express the receptor for this cytokine, CD127. In fact, CD127 has been used as a critical marker to identify human ILCs (Vivier et al., 2018). Whereas CD127^+^ ILC2s are readily detected in most human fetal and adult tissues, they are rare in adult intestine (Forkel et al., 2019). We have now found ILC2s within the CD127^−^ compartment, suggesting that in the adult human intestine, ILC2s mostly lack CD127. It may be possible that CD127^+^ ILC2s, which are abundant in the fetal intestine (Mjösberg et al., 2012), modify their phenotype by downregulating the expression of CD127 after birth following interactions with the gut microbiome or products from food digestion. Interestingly, CD127^+^ ILC2s are abundant in the intestine of adult mice. Whether this is a consequence of being housed in specific pathogen–free conditions should be addressed in future studies.

Memory ILC2s are characterized by transcriptional reprogramming toward cell activation and enrichment for MAPK pathway members, such as *FOS* and *JUN*, which are downstream effectors of p38 activation. Importantly, p38 MAPK has been shown to be a central pathway of memory T helper 2 cells to respond to IL-33 in mouse and human (Endo et al., 2015). It is likely that activated ILC2s downregulate CD127 expression as they become CD45RO^+^ memory cells. In this context, it is tempting to speculate that memory ILC2s remain in tissues by relocating to IL-7–rich environments, therefore explaining the downregulation of CD127. This would explain why in PB of some individuals CD127^+^CD45RO^+^ ILC2s can be found, where these tissue signals are likely absent. We cannot exclude the possibility that some memory ILC2s have stably downregulated CD127, especially in the adult human intestine where CD127^+^ ILC2s are rarely present.

Zwijnenburg et al. (2023) have recently suggested that T cell differentiation occurs as a continuum of cellular states rather than transitioning between discrete subsets. They identified CX3CR1 as a graded marker for functionally distinct states of human antigen–experienced CD8^+^ T cells. It is likely that naive, activated, and memory ILC2s can also be identified as a continuum of cellular states rather than distinct subsets. The inverse correlation of CD45RO and CD127 expressions on ILC2s in the nasal polyps indicates that CD127 expression gradient on ILC2s may reflect the broad spectrum of cell state on ILC2s.

Downregulation of CD127 on IL-33–activated mouse ILC2s has been previously suggested (Entwistle et al., 2020; Li et al., 2017). We now show that this low expression of CD127 on ILC2s is maintained as they acquire immunological memory and that CD127^−^ memory ILC2s are the major producers of IL-5 and IL-13 upon rechallenge. Naive mice also have CD127^−^ ILC2s, suggesting that these cells have a history of being activated. Indeed, previous reports (Schneider et al., 2019) have shown that after weaning a transient wave of IL-33 in peripheral tissues prime ILC2s, which then increase expression of *Nr4a1*, which is associated with heightened responsiveness of ILC2s. Interestingly, our data shows that *NR4A1* expression is significantly higher in skin CD127^−^ ILCs from healthy donors as compared with CD45RA^+^ ILC2s.

Finally, we have also identified Lin^−^CD127^−^CRTH2^−^CD200R1^+^CD45RO^+^ cells within the c-KIT^+^ and c-KIT^−^ gates (data not shown), suggesting the existence of memory ILC1 and ILC3. Of note, mouse memory ILC3 has been recently described (Serafini et al., 2022). It may be possible that the Lin^−^ CD127^−^CRTH2^−^CD200R1^+^CD45RO^+^ population also contains memory ILC2s, which have downregulated CRTH2, as it has been shown that CRTH2^−^ ILCs from human PB and lung cells can produce IL-5 (Liu et al., 2020).

Our results highlight the need for a re-evaluation of the established gating strategies used to identify and isolate human ILCs in various tissues, as the ability of ILC2s to regulate the expression of CD127, a well-recognized marker for human ILCs, has become evident. Instead, a combination of CD127 with the expression of CD45RA and CD45RO may offer insights into the cellular state of ILC2s. Alternatively, the CD200R1 marker, in conjunction with CD161, emerges as a promising candidate that could improve our gating strategy for defining human ILC2s within tissues. Furthermore, the discovery of highly responsive CD127^−^CD45RO^+^ ILC2s in inflamed nasal polyps from patients with CRSwNP suggests the potential relevance of memory ILC2s in this and other type 2 diseases. In line with this, our recent observations indicate that the presence of CD45RO^+^ ILC2s correlates with a quicker clinical response among patients with chronic CRSwNP receiving treatment with the anti-IL-4Ra antibody dupilumab (Golebski et al., 2023). Immunological memory of ILC2s may have important implications in allergic diseases, especially in the so-called non-allergic asthma (Papi et al., 2018), as, in contrast to T cells, ILC2s respond in an antigen-non-specific manner. Therefore, it may explain why some allergic patients react to unrelated allergens.

Whether experienced ILCs should be referred to as memory or trained is currently under debate (Hartung and Esser-von Bieren, 2022; Martinez-Gonzalez et al., 2018, 2017). The term trained immunity has been associated with epigenetic reprogramming of myeloid cells that confers them with increased fitness to respond to infections. Although there may be similarities in the concept with lymphoid memory, we argue that the training observed in macrophages and neutrophils may not be as enduring as the memory seen in ILCs, NK cells, and T/B cells.

Both memory ILC2s and memory T cells are in a resting state and capable of responding stronger upon secondary challenge, and both are phenotypically distinct from their naive counterpart. Memory ILC2s do not only react to the initial priming agent but can also respond more intensely to unrelated stimuli. This is comparable to memory T cells that can respond better than naive T cells to crossreacting antigens different from the priming antigen and to bystander activation (Benichou and Kim, 2017; Lechler and Lombardi, 1991).

## Materials and methods

### Human tissue collection

Healthy skin tissues were obtained as excess materials from plastic surgery of the breast or abdomen. Intestinal samples were obtained after surgical resection with the exclusion of subjects that had undergone chemo- or radiotherapy prior to surgery. Fetal intestine was obtained from aborted fetuses at the Stichting Bloemenhoven clinic in Heemstede, Netherlands. Gestational age ranged from 14 to 19 weeks, which was determined by ultrasonic measurement of the skull or femur diameter. Lung tissues were obtained from excess materials from lobectomy surgery of non-small cell lung cancer at Erasmus Medical Center Rotterdam. Nasal polyp samples were collected from patients with CRSwNP undergoing endoscopic sinus surgery. Buffy coat was collected from healthy volunteers recruited by the blood bank at the Sanquin Blood Bank and Karolinska University Hospital. The collection and use of all human samples was approved by the Medical Ethical Committee of the Amsterdam University Medical Center and Erasmus University Medical Center and with informed consent.

### Isolation of cells from human samples

Single-cell suspension was obtained from intestinal tissues (adult and fetal) and skin as previously described (Bernink et al., 2019; Krabbendam et al., 2018b, 2021). Nasal tissues were processed as preciously described (Krabbendam et al., 2018b). Lung tissue was cut into small pieces, blood was washed away with PBS, and tissue pieces were digested in RPMI + Collagenase D (1 mg/ml) + DNase I (0.1 mg/ml) for 1 h. Peripheral blood mononuclear cells were isolated from buffy coats as previously described (Tindemans et al., 2019).

### Mice

C57BL/6J (B6) mice were bred in the British Columbia Cancer Research Centre animal facilities or purchased at Karolinska Institute animal facilities from the Jackson and Janvier Laboratory. RORα-YFP mice were generated in-house as previously described (Ghaedi et al., 2020). All animal use was approved by the animal care committee of respective institutions and mice were maintained in a specific pathogen–free facility. Briefly, mice were housed in vented cages (four mice maximum per cage) with crinkle paper or cotton nesting materials and a hiding place. They were fed a normal chow diet. Mice were anesthetized by isoflurane inhalation during i.n. treatment to minimize animal suffering and distress. After the injections, they were moved to a separate cage placed on a heating pad and monitored closely until they were fully recovered from anesthesia. The health status of the mice was assessed daily during the period of the treatments and 1 day after the last injections based on their appearance and behavior. At the time of the harvest, mice were anesthetized by isoflurane inhalation until they were unconscious and euthanized by carbon dioxide asphyxiation. All mice were used between 6 and 12 weeks of age.

### Tissue processing and primary leukocyte preparation from mice

Lungs were collected from mice and chopped into small pieces using a razor blade. Pieces of lungs were enzymatically digested in 5 ml of DMEM + 10% FBS and supplemented with 118.05 Kunitz U/ml DNase I and 142.5 U/ml collagenase IV (37°C, 25 min, 250 rpm shaker). Digested lungs were mashed through a 70-μm strainer and washed with 5 ml DMEM + 10% FBS, after which they were centrifuged (5 min, 400 *g*) and the supernatant was discarded. Leukocytes were isolated by density gradient centrifugation (10 min, 650 *g*, no acceleration or brake) in 5 ml of 36% Percoll solution. Red blood cells were lysed in 3 ml of 150 mM ammonium chloride solution and washed with 5 ml PBS + 2% FBS.

For preparation of single-cell suspension from the small intestine, Peyer’s patches were removed and the intestine was fragmented into three to four pieces. Each piece was longitudinally cut open and washed in 5 ml wash buffer (88.5% water, 10% of 10× Hanks’ Balanced Salt [HBSS] solution, and 1.5% of 1 M HEPES, pH = 7.2) until clean. The cleaned pieces were further cut into 1 cm and washed in the wash buffer by vigorously shaking three times, after which they were resuspended in 20 ml of prewarmed (37°C) intestinal epithelial fraction buffer (77.4% water, 10% of 10× HBSS, 10% FBS, 1.5% of 1 M HEPES, 1% of 0.5 M EDTA, and 0.1% of 1 M dithiothreitol, pH = 7.2) and incubated while shaking (37°C, 20 min, 250 rpm shaker). The buffer was removed after vigorous shaking and samples were washed three times in wash buffer as above. The incubation step was repeated and the residual intestine pieces were resuspended in 20 ml lamina propria buffer (88.4% RPMI 1640 with HEPES and without glutamine, 10% FBS, 1.5% of 1 M HEPES, and 0.1% of 1 M dithiothreitol, pH 7.2) and supplemented with 100 U/ml collagenase VIII (C2139; Sigma-Aldrich) and 9.41 Kunitz U/ml DNase I. Samples were incubated at 37°C for 1 h in a shaker at 250 rpm, after which the supernatant was collected through a 70-μm strainer. The incubation step was repeated for the second time, and the cells were collected by centrifuging the combined flow through (4°C, 15 min, 400 *g*). Cells were resuspended in 5 ml 90% Percoll, which was overlaid with 8 ml of 36% Percoll, and subjected to density gradient separation (20 min, 650 g, no acceleration or brake). Leukocytes were isolated by collecting the interphase and washed with 12 ml PBS + 2% FBS and pelleted by centrifugation (4°C, 10 min, 400 *g*).

For single-cell suspension of skin cells, both ears were collected from mice and split into dorsal and ventral halves using forceps. Ear tissues were digested in 5 ml PBS + 2% FBS containing 420 U/ml collagenase IV (37°C, 40 min, 250 rpm shaker). Digested skin was then mashed through 70-μm strainers, followed by washing with 5 ml PBS + 2% FBS. Cells were collected by centrifugation (4°C, 5 min, 400 *g*) and washed again with 10 ml PBS + 2% FBS.

### Antibodies, reagents, and flow cytometers

Isolated leukocytes were counted using a hemocytometer and incubated in 2.4G2 mAb to block Fc receptors for analyses by FACS. Fluorescein isothiocyanate (FITC)–conjugated anti-mouse KLRG1 (2F1), PerCP-Cy5.5-conjugated anti-mouse CD25 (PC61.5), PerCP-eFluor 710–conjugated anti-mouse T1/ST2 (RMST2-2), rat IgG2a, κ isotype control (eBR2a), allophycocyanin (APC)-conjugated anti-mouse KLRG1 (2F1), eFluor 660–conjugated anti-mouse/human GATA3 (TWAJ), AF700-conjugated anti-mouse CD127 (A7R34), eFluor 450–conjugated CD11b (M1/70), CD11c (N418), CD19 (1D3), CD3ε (145-2C11), CD4 (RM4-5), Gr-1 (RB6-8C5), NK1.1 (PK136), TCRβ (H57-597), TCRγδ (GL3), Ter119 (TER-119), PE-conjugated anti-mouse IL-13 (eBio13A), anti-mouse CD127 (A7R34), anti-mouse/human GATA3 (TWAJ), anti-mouse RORγt (B2D), rat IgG2a, κ isotype control (eBR2a), PE-Texas Red–conjugated anti-human CD45RA (MEM-56), PECy7-conjugated anti-mouse CD127 (A7R34), anti-mouse/human Tbet (eBio4B10), anti-human IL-22 (22URTI), PECy5-conjugated anti-mouse CD25 (PC61.5) were purchased from Thermo Fisher Scientific. FITC-conjugated anti-mouse CD69 (H1.2F3), anti-mouse Ter119 (TER-119), APC-conjugated anti-mouse/human IL-5 (TRFK5), rat IgG1, κ isotype control (R3-34), APCCy7-conjugated anti-mouse CD90.2 (53-2.1), AF700-conjugated anti-human CD45 (HI30), BV421-conjugated anti-human CD200R1 (OX-108), anti-human IL-13 (JES10-5A2), V500-conjugated anti-mouse CD45 (30-F11), BV510-conjugated anti-human CD45RO (UCHL1), anti-mouse CD11b (M1/70), BV605-conjugated anti-human CD25 (2A3), BV605-conjugated anti-mouse T1/ST2 (U29-93), BV650-conjugated anti-human ICOS (DX29), BUV395-conjugated anti-mouse NK1.1 (PK136), BUV496-conjugated anti-mouse CD4 (GK1.5), BUV737-conjugated anti-mouse CD11c (HL3), BUV805-conjugated anti-mouse Ly6g (1A8), PE-conjugated anti-human CD25 (M-A251), anti-human CD161 (DX12), anti-mouse CD44 (IM7), PECF594-conjugated anti-human CRTH2 (BM16), and PECy5-conjugated anti-human CD117 (YB5.B8) were purchased from BD Biosciences. FITC-conjugated anti-human CRTH2 (BM16), CD1a (HI149), CD3ε (OKT3), CD4 (RPA-T4), CD14 (HCD14), CD19 (HIB19), CD34 (581), CD123 (6H6), TCRαβ (IP26), TCRγδ (B1), FcεRIα (AER-37 (CRA-1)), BDCA2 (201A), CD56 (HCD56), AF700-conjugated anti-human CD3ε (UCHT1), APCCy7-conjugated anti-human CD45 (2D1), CD45RO (UCHL1), BV421-conjugated anti-human CD127 (A019D5), BV 510–conjugated anti-human CD161 (HP-3G10), Pacific Blue–conjugated anti-mouse FcεR1α (MAR-1), BV570-conjugated anti-mouse Ly6c (HK1.4), BV605-conjugated anti-mouse CD90.2 (53-2.1), PE-conjugated anti-mouse/human KLRG1 (2F1/KLRG1), rat IgG1, κ isotype control (RTK2071), anti-mouse/human CD45R/B220 (RA3-6B2), PE/Dazzle 594–conjugated anti-human CD200R1 (OX-108), PECy7-conjugated anti-human CD127 (A019D5), rat IgG2a, κ isotype control (RTK2758) were purchased from BioLegend. FITC-conjugated anti-mouse T1/ST2 (DJ8) was purchased from MD Bioproducts. BV605-conjugated anti-human CD45 (HI30) was purchased from Sony. PECy5.5-conjugated anti-human CD117 (104D2D1) was purchased from Beckman Coulter. BD LSRFortessa and SONY ID7000 were used for flow cytometric analyses. Samples were first gated on live cells using eFluor 780 fixable viability dye or Zombie-NIR fixable viability dye, followed by single-cell gating. Mouse lineage cocktail contains CD3ε, CD4, CD19, TCRβ, TCRγδ, CD11b, CD11c, NK1.1, Ter119, and Gr-1, while human lineage cocktail contains CD1a, CD3ε, CD4, CD5, CD14, CD16/32, CD19, CD34, CD123, TCRαβ, TCRγδ, FcεRIα, BDCA2, CD8α, CD11c, and CD56. FlowJo version 10.0.7r2 and 10.0.0 was used for data analyses.

### In vitro culture

For in vitro culture of human ILC2s, purified cells were plated in a 96-well round-bottom plate in Yssel’s media (IMDM + 4% vol/vol Yssel’s supplement [made in-house, Amsterdam University Medical Center] + 1% vol/vol human AB serum [Invitrogen]). ILC2s were cultured in the presence of IL-2 (10 U/ml or 20 ng/ml) plus IL-7, and different combinations of IL-25, IL-33, TSLP, IL-1β, and IL-18 (20 or 40 ng/ml), specified in the figure legends. Cytokines were supplemented every 3 days. IL-5 was detected by intracellular staining and flow cytometry.

### Quantitative real-time PCR

Purified cells were lysed, and RNA was isolated using the NucleoSpin RNA XS kit (Macherey-Nagel) according to the manufacturer’s protocol. Complementary DNA was synthesized using a High-Capacity Archive kit (Applied Biosystems). qPCR was performed with LightCycler 480 Instrument II (Roche) with SYBR Green I master mix (Roche).

### CEL-Seq2–based scRNAseq

For scRNAseq, ILCs were purified from the dermis of healthy skin. Isolation was performed directly after surgery. 1,583 cells were directly sorted in 384-well plates, frozen, and sent to Single Cell Discoveries for further processing of their pipeline based on CEL-Seq2 (Muraro et al., 2016). Libraries were run on a HiSeq4000 for Illumina sequencing. After processing and quality control (QC) were performed by Single Cell Discoveries. Reads were aligned to GRCh38 reference assembly using STARsolo (Dobin et al., 2013). All transcriptome data analysis was performed using Seurat package v4. Data QC was performed to exclude ERCC genes from the count matrix. Cells with <500 genes and a high percentage of mitochondrial genes were removed from downstream analyses. The ratio of the number of genes over the number of UMIs was over 0.85 (novelty score). Following data QC, we obtained a matrix with 1,449 cells and 15,401 genes. Data normalization was performed using the sctransform within Seurat analysis pipeline, removing mitochondrial genes as a confounding source of variation. Dimensionality reduction was performed using principal component analysis, clustering was done by shared nearest neighbor approach and Louvain algorithm, and visualization was performed using UMAP. Differential gene expression analysis was determined using a Wilcoxon Rank Sum test approach as implemented in Seurat. Pathway enrichment analysis was performed in R using the enrichR package. Adjusted P values were calculated using Bonferroni correction. Mean fluorescence intensity (MFI) values for cell surface markers were obtained from index sort data and added as a new assay to the Seurat object.

### Single-cell cloning

3,000 per well of OP9-DL1 murine stromal cells were plated in a 96-well round-bottom plate at least 5 h prior to the start of the culture. CD127^+^ and CD127^−^ ILC2s were sorted as single cells per well in the 96-well round-bottom plate and were expanded in Yssel’s medium supplemented with 1% (vol/vol) human AB serum in the presence of IL-23, IL-1β, and TGF-β (all at a concentration of 20 ng/ml). Clones were analyzed within 14 days of culture.

### Cell staining for FACS analyses

Cells isolated from tissues were stained with surface stains for half an hour at 4°C, followed by FACS analyses or intracellular staining. For intracellular cytokine staining of murine samples, leukocytes were incubated at 37°C for 3 h in 500 μl RPMI 1640 media containing 10% FBS, 100 U/ml penicillin/streptomycin, 50 μM 2-ME, Brefeldin A (GolgiPlug; BD Biosciences), 30 ng/ml PMA (P1585; Sigma-Aldrich), and 500 ng/ml ionomycin (10634; Sigma-Aldrich). Human cells were stimulated 3 h with PMA (50 ng/ml) and ionomycin (500 nM) with GolgiStop. Intracellular cytokine staining was performed after pre-incubation and surface staining using Cytofix/Cytoperm Fixation/Permeabilization Solution kit (BD Biosciences) according to the manufacturer’s protocol. CellTrace Violet (CTV) staining was performed according to the manufacturer’s protocol. Transcription factor staining was performed without preincubation using Foxp3/Transcription Factor Staining Buffer Set (Thermo Fisher Scientific) according to the manufacturer’s protocol. For intracellular cytokine and intranuclear staining of RORα-YFP mice, cells were prefixed with 0.5% paraformaldehyde for 5 min at room temperature, before proceeding to fixation, permeabilization, and intracellular staining.

### In vivo stimulation of mice

Mice were anesthetized by isoflurane inhalation and given i.n. administrations of 0.25 µg IL-33 (BioLegend) or 0.218U papain (Sigma-Aldrich) in 40 μl PBS.

### Contact for reagent and resource sharing

Further information and requests for resources and reagents should be directed to and will be fulfilled by corresponding author Itziar Martinez-Gonzalez.

### Online supplemental material

Fig. S1 shows scRNAseq analyses of nasal polyps from CRSwNP patients and biopsies from AD patients. Fig. S2 shows ILC2 expression of CD200R1 and scRNAseq analyses of healthy dermis. Phenotypes of CD127^+^ and CD127^−^ ILC2s and the analyses of single-cell clones derived from CD127^+^ and CD127^−^ ILC2s are also presented. Fig. S3 shows gene and protein expressions of various clusters of ILCs and the responsiveness of CD45RA^+^ and CD45RO^+^ human ILC2s upon in vitro stimulation. Fig. S4 shows the absence of T cell contamination in ILC2 gate and alternative gating strategies to identify CD127^+^ and CD127^−^ ILC2s in mice. Fig. S5 shows the phenotype of in vitro–activated human ILC2s and analyses of in vitro–generated memory ILC2s.

## Data Availability

The data reported in this paper have been uploaded to the Gene Expression Omnibus with the accession number GSE267098.
